# Macrophage-specific therapy blocks the lung’s mechanosensitive immune response to alveolar distension

**DOI:** 10.1172/jci.insight.191853

**Published:** 2025-10-30

**Authors:** Liberty Mthunzi, Mohammad N. Islam, Galina A. Gusarova, Brian Karolewski, Sunita Bhattacharya, Jahar Bhattacharya

**Affiliations:** 1 Lung Biology Laboratory; Division of Pulmonary, Allergy, and Critical Care Medicine; Department of Medicine; Vagelos College of Physicians and Surgeons; and; 2 Institute of Comparative Medicine, Columbia University Irving Medical Center, Columbia University, New York, New York, USA.

**Keywords:** Cell biology, Inflammation, Pulmonology, Calcium signaling, Macrophages

## Abstract

The lung’s mechanosensitive immune response to alveolar overdistension impedes ventilation therapy for hypoxemic respiratory failure. Though mechanistically unclear, the prevailing hypothesis is that the immune response results when alveolar overdistension stretches alveolar macrophages (AMs). Since this hypothesis is untested in live lungs, we optically imaged live mouse alveoli to detect alveolus-adherent, sessile AMs that communicate with the alveolar epithelium through connexin 43-containing (Cx43-containing) gap junctions. Alveolar hyperinflation did not stretch the AMs, but it increased AM Ca^2+^. AM-specific *Cx43* deletion blocked the Ca^2+^ response, as well as lung injury due to mechanical ventilation at high tidal volume (HTV). HTV-induced injury was also inhibited by AM-targeted delivery of liposomes containing the inhibitor of endosomal Ca^2+^ release, xestospongin C. We conclude Cx43- and Ca^2+^-dependent AM-epithelium interactions determine the lung’s mechanosensitive immunity, providing a basis for therapy for ventilator-induced lung injury.

## Abstract 2

Gap junction dependent calcium signaling between alveolar epithelium and alveolar macrophages underlies the lung’s mechanosensitive immunity due to hyperinflation.

## Graphical abstract

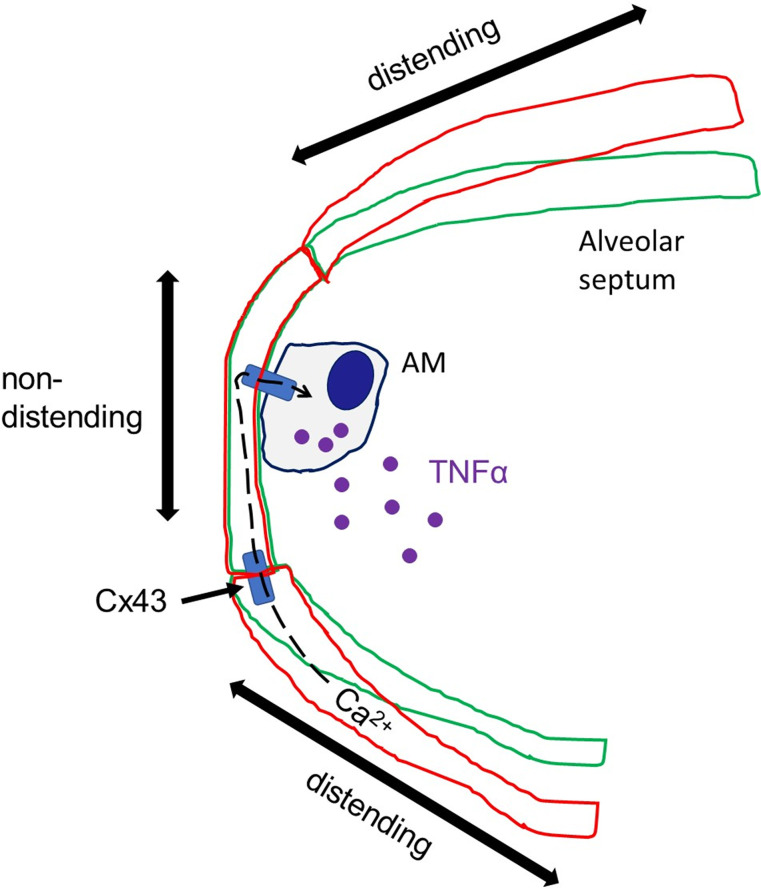

## Introduction

Mechanical ventilation (MV), critical therapy in hypoxemic acute lung injury (ALI), which leads to acute respiratory distress syndrome (ARDS) (1), supports blood oxygenation and lung recovery. However, mechanical ventilation is itself pro-inflammatory in that it can cause a mechanosensitive immune response, resulting in injury to the air-blood barrier, which reverses the protective effects of ventilator therapy and contributes to morbidity and mortality in ARDS (2, 3). It is proposed that mechanical injury results from alveolar overexpansion during MV (3, 4). However, the mechanistic link between alveolar overexpansion and the immune response remains poorly defined.

According to the prevailing hypothesis, alveolar overexpansion causes stretch of alveolar macrophages (AMs), thereby activating AM-initiated lung inflammation (5). Implicit here is the notion that the alveolar wall and AMs are mechanically coupled such that wall strain due to alveolar expansion concomitantly induces AM stretch, and that the stretch activates pro-inflammatory signaling. However, supportive evidence for these mechanisms is lacking in live lungs. Thus, although in vitro evidence indicates that cell stretch evokes Ca^2+^ influx in cultured macrophages (6, 7), there is no evidence in intact alveoli that AMs are mechanically stretched or that they evoke pro-inflammatory Ca^2+^ responses during alveolar expansion.

This understanding is complicated by the fact that alveolar expansion occurs nonuniformly in that some regions of the alveolar wall are strain protected and distend little relative to other regions, which are strain sensitive (8). Hence, wall stretch might be better transmitted to AMs at strain-sensitive than strain-protected sections during alveolar expansion. A direct test of this hypothesis is possible by real-time confocal microscopy (RCM) of the intact lung, since superimposition of alveolar images obtained at baseline and expansion reveals the pattern of alveolar strain distribution (8). Here, we applied these approaches to test the AM stretch hypothesis through quantification of AM shape responses to alveolar overexpansion.

For translational relevance, we determined alveolar expansion patterns in live human lungs as we described previously (9). Since human lungs are in relatively short supply, we also determined responses in pristine pig lungs, which provide a translational model for studies of mechanical ventilation (10, 11). An associated goal was to define the extent to which hyperinflation activated Ca^2+^ influx in the AMs, thereby priming the pro-inflammatory response. We report below our unexpected findings that resident AMs were located in strain-protected alveolar niches, so they did not stretch during alveolar expansion. Nevertheless, Ca^2+^ communication through connexin 43–containing (Cx43-containing) gap junctions between the stretched alveolar epithelium and nonstretched AMs determined the lung’s mechano-immunity. Based on these and other findings, we propose a therapeutic approach for suppressing this signaling and hence the pro-inflammatory response to alveolar overexpansion.

## Results

### Alveolar expansion does not change cell shape in AMs.

**Figure 1:**
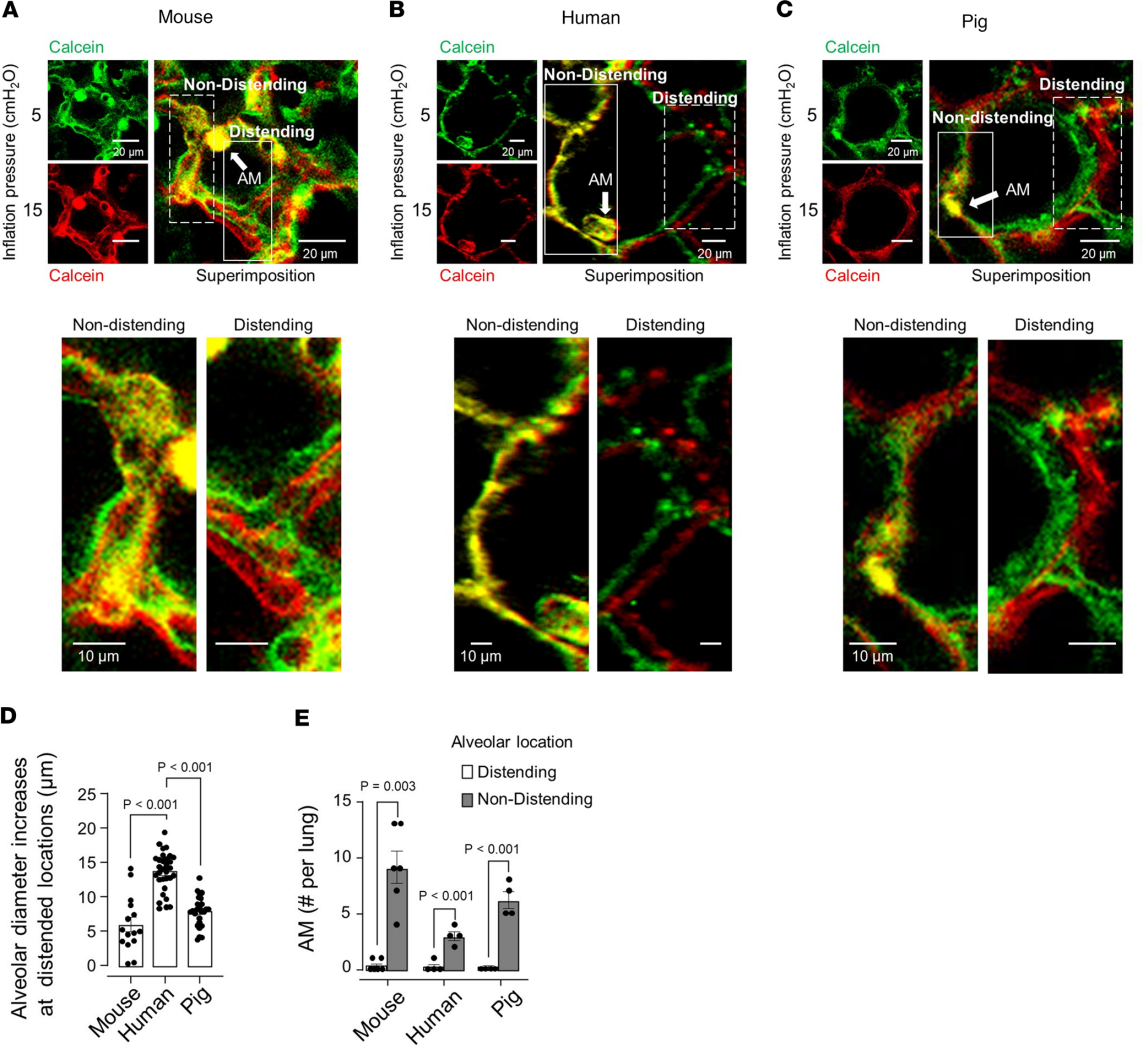
Pseudocolor superimposition by confocal microscopy reveals AMs are located in strain-protected alveolar niches. (**A**–**C**) Representative confocal images of mouse (**A**), human (**B**), and pig (**C**) alveoli are shown at low (left images) and high magnification (right images). Images were obtained at the indicated inflation pressures. The low-magnification images are pseudocolored differently for the different inflation pressures. The merge image was obtained by superimposing the green and red images. The superimposition reveals yellow pseudocolor at a nondistending alveolar segment (solid rectangle) but separation of red and green at the distending segment (dashed rectangle). Images were centered for superimposition. An AM is located at the corner of the nondistending alveolar segment (arrow). High-magnification images of rectangles are below. (**D**) Bars show hyperinflation-induced alveolar diameter increase at sites of alveolar distension in 4 lungs each for mouse, human, and pig. Findings were replicated in at least 3 alveoli in each lung. (**E**) Bars show numbers of AMs at distending and nondistending locations of alveoli in 4 lungs each for mouse, human, and pig. Each dot represents data for 1 lung. Data analyses. Group data are mean ± SEM. *P* values were calculated by 1-way ANOVA with Bonferroni’s correction (**D**) or paired *t* test (**E**).

To determine the effects of alveolar expansion, we viewed alveoli in mouse, pig, and human lungs by RCM. Alveolar microinfusions of calcein-AM (calcein) induced alveolar epithelial fluorescence, marking the alveolar perimeter (Figure 1, A–C). To map the strain pattern of the alveolar wall, we applied our reported approach by which we imaged an alveolus at low and high inflation pressures, assigning a different pseudocolor for each image (8). Superimposition of the images reveals congruence or separation of the pseudocolors at, respectively, nondistended (strain protected) or distended (strain sensitive) alveolar segments. We increased inflation pressure from 5 to 15 cmH_2_O, a procedure that increases lung volume to about 80% of total lung capacity (8). We avoided very high airway pressures, such as those applied in previous studies (12), to eliminate the risk of disruptive alveolar injury and bleeding.

We imaged alveoli at multiple optical planes along the depth axis; then we compared images at the alveolar equator. These analyses indicated that at the level of single alveoli, increase of inflation pressure caused uneven alveolar distension not only in mouse lungs, as we reported (8), but also in human and pig lungs (Figure 1, A–C). Thus, we detected strain-sensitive locations at which the alveolar diameter increased by several micrometers (Figure 1D), while concomitantly strain-protected regions were also present at which there were no diameter changes.

We identified AMs by the alveolar immunofluorescence (IF) of specific markers (Supplemental Figure 1A; supplemental material available online with this article; https://doi.org/10.1172/jci.insight.191853DS1). We used IF of SiglecF and CD11c for mouse AMs (13), of Siglec1 for human AMs (14, 15), and of CD203a for pig AMs (16). Alveolar diameter was larger in human than in pig or mouse alveoli (Supplemental Figure 1B) (17). Hence, in a single imaging field, we viewed approximately 40 mouse or pig alveoli but only 8–10 human alveoli. Despite the differences in alveolar size, for every 10 alveoli we detected 2–5 AMs in all species (Supplemental Figure 1C). The AMs did not move after hyperinflation (Supplemental Figure 1D), indicating they were sessile. Our finding was that sessile AMs were exclusively located at strain-protected alveolar regions that did not distend after hyperinflation (Figure 1E).

To determine hyperinflation-induced changes in AM morphology, we carried out imaging at different optical levels down the depth axis of AMs at each inflation pressure, as shown in the sketch (Supplemental Figure 2A). To assess cell shape changes, we analyzed the images by pseudocolor superimposition to assess cell strain and by quantifications of cell diameter and cell circumference. The diameters were assessed on the principle that the AM shape approximates a spheroid, and hence diameters in the minor and major axes define shape, and that shape distortion is defined by the relative displacement of these axes between adjacent transverse planes (18).

**Figure 2:**
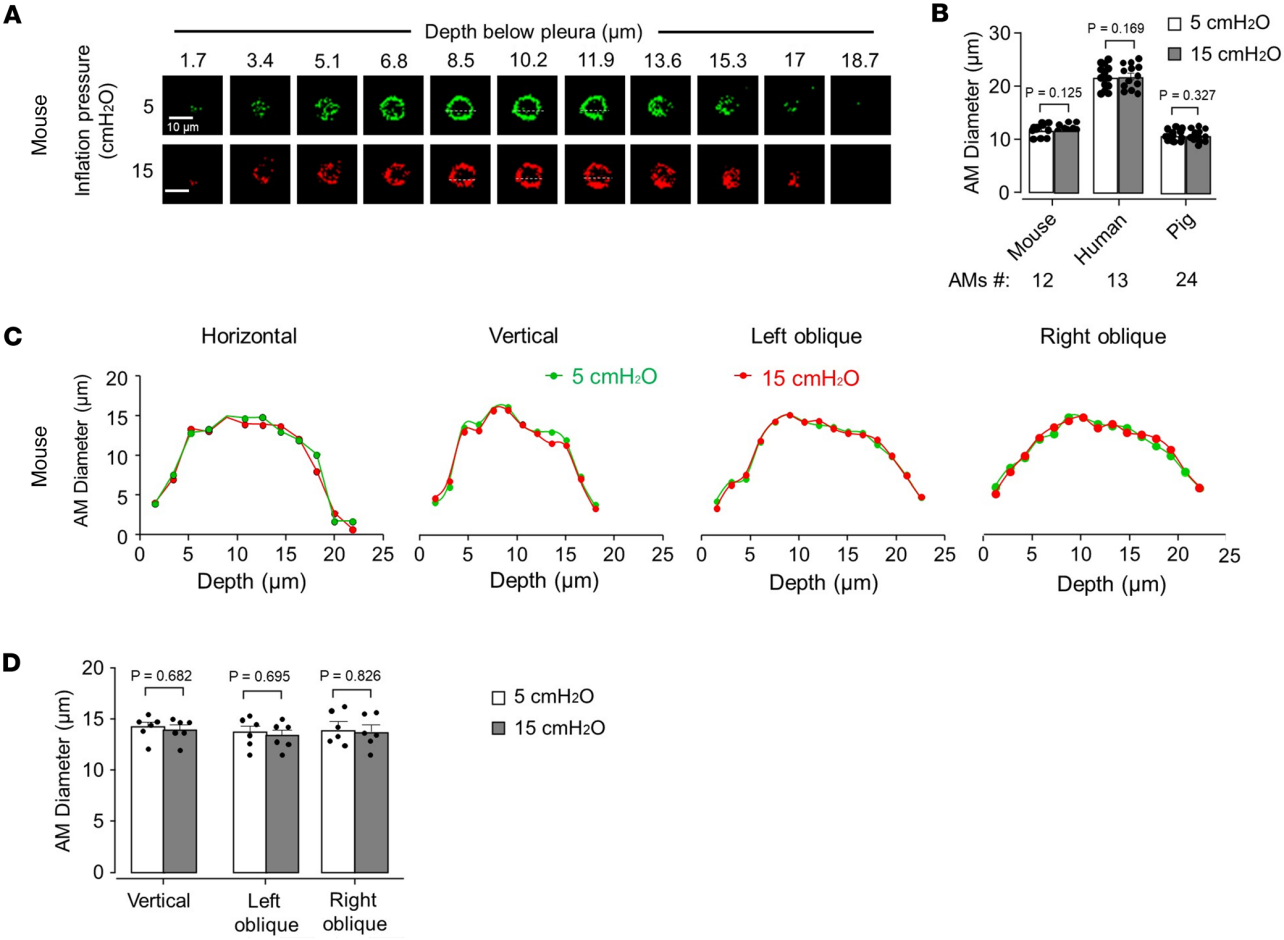
Alveolar expansion does not change AM shape during mouse, human, and pig lung hyperinflation. (**A**) Confocal *z* stack images show mouse (SiglecF^+^) AM perimeter at 5 (green) and 15 (red) cmH_2_O inflation pressure at different depths. Replicated 13 times in 4 lungs. (**B**) Bars show paired diameter quantifications in the horizontal axis for single macrophages (dots) at the indicated inflation pressures in 4 lungs each for mouse, human, and pig. (**C** and **D**) The tracings (**C**) plot mouse AM diameter along the indicated axes at different depths below the pleura. Replicated 6 times in 4 lungs. Bars (**D**) show diameter quantifications in the indicated axes for single macrophages (dots) at 2 inflation pressures in mouse lungs. Data analyses. Group data are mean ± SEM. *P* values were calculated by paired *t* test (**B** and **D**).

As shown by images for single experiments in mouse (Figure 2A), pig, and human (Supplemental Figure 2, B and C) lungs, and in the group data (Figure 2B), human AMs were almost 2 times larger than mouse and pig AMs, as determined by diameter measurements at the cell equator. Fluorescence of the membrane marker for human AMs often appeared to partially fill the space inside the cell perimeter. This is depicted, for example, for the optical slices taken at 10.2–18.7 micrometers below the pleura (Supplemental Figure 2C). Since Siglec fluorescence is cell membrane specific, this pattern of fluorescence distribution may have occurred because the cell membrane folded, such that the membrane fold lay over the cell interior, causing the fold’s Siglec1 fluorescence to falsely appear as “cytosolic.” The membrane folding may have occurred more in human AMs than in pig or mouse AMs because they were larger. The possibility that the marker was internalized in the cell may be ruled out as other optical sections of the same cell, as for example, the ones at depths of 5.1–8.5 confirmed that the marker was localized to the cell periphery.

For each species global cell shape responses to hyperinflation were similar. Thus, superimposed pseudocolors obtained at different inflation pressures were completely merged for all AMs (Supplemental Figure 2, D–F), suggesting that hyperinflation did not detectably change cell shape at any optical level. Further, to the extent that there were no changes in SiglecF and CD11c (Supplemental Figure 2, G and H), we interpret that hyperinflation did not cause detectable changes in cell surface markers of AMs.

We plotted AM diameters at the vertical, horizontal, and oblique axes as shown in the sketch (Supplemental Figure 2I) against optical depth with the expectation that shape changes due to hyperinflation would be reflected by noncongruency of the plots. However, contrary to this expectation, the plots for all diameters were completely congruent for the AMs (Figure 2, C and D, and Supplemental Figure 2, B–F). Although hyperinflation caused alveolar expansion, AM circumferences remained unchanged at all levels in the depth axis (Supplemental Figure 2, J–L). Taken together, these findings indicate that AMs did not change cell shape; hence, they did not stretch during alveolar overexpansion.

### Hyperinflation increases Ca^2+^ in AMs.

**Figure 3:**
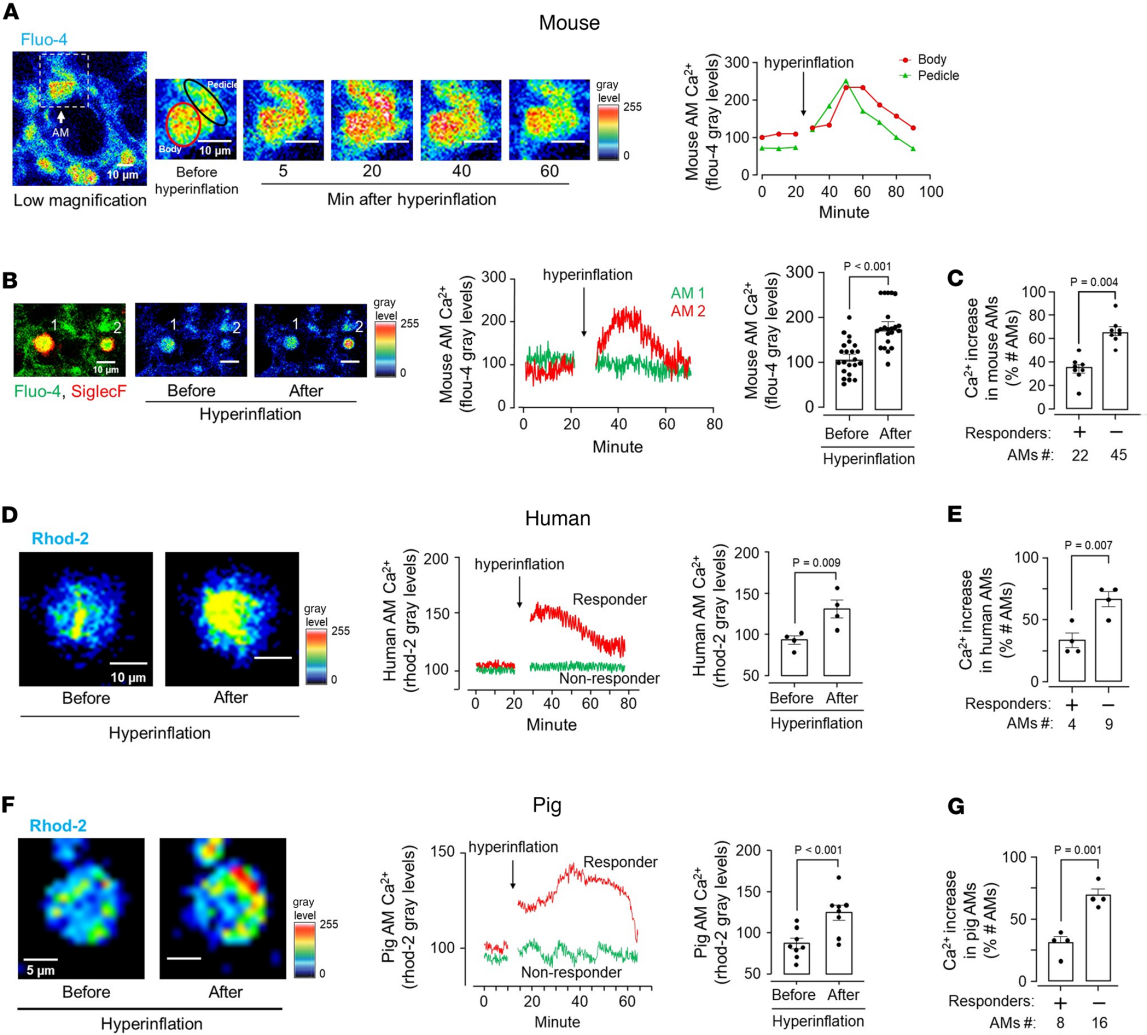
Hyperinflation causes calcium mobilization in mouse, human, and pig sessile AMs. (**A**) Confocal images of mouse alveolar epithelium loaded with the fluorescent cytosolic calcium indicator, fluo-4, show cytosolic Ca^2+^ fluorescence in a single AM at low (rectangle in low-magnification image on the left) and high magnification before (second image from the left) and after hyperinflation (third–sixth images from the left). Pseudocolors represent fluorescence expressed as gray levels as defined in the symbol key. Tracings show Ca^2+^ in cell body and pedicle (red and black circles in second image from the left). Replicated in 4 lungs. (**B**) Images from adjoining alveoli show AMs that responded with hyperinflation-induced Ca^2+^ increase (AM #2) versus an AM that did not respond (AMs #1). Tracings show corresponding time courses. Bars show comparisons of maximum Ca^2+^ before and after hyperinflation in AMs that responded to hyperinflation. Data are for 22 AMs from 6 mouse lungs. (**C**) Bars show comparisons for responders (+) and nonresponders (–) from mouse lungs. (**D**) Confocal images of human AMs loaded with the fluorescent mitochondrial calcium indicator, rhod-2, show mitochondrial Ca^2+^ fluorescence in a single AM at high magnification before and after hyperinflation. Pseudocolors represent fluorescence expressed as gray levels as defined in the symbol key. Tracings show mitochondrial Ca^2+^ in AMs. Bars show comparisons of maximum Ca^2+^ before and after hyperinflation in AMs that responded to hyperinflation. Data are for 4 AMs from 4 human lungs. (**E**) Bars show comparisons for responders (+) and nonresponders (–) from human lungs. (**F**) Confocal images of pig AMs loaded with the fluorescent mitochondrial calcium indicator, rhod-2, show mitochondrial Ca^2+^ fluorescence in a single AM at high magnification before and after hyperinflation. Pseudocolors represent fluorescence expressed as gray levels as defined in the symbol key. Tracings show mitochondrial Ca^2+^ in AMs. Bars show comparisons of maximum Ca^2+^ before and after hyperinflation in AMs that responded to hyperinflation. Data are for 8 AMs from 4 lungs. (**G**) Bars show paired comparisons for responders (+) and nonresponders (–) from pig lungs. Data analyses. Group data are mean ± SEM. *P* values were calculated by paired *t* test (**B**–**F**).

To interrogate hyperinflation-induced AM mechanisms that occurred despite the absence of cell stretch, we quantified AM Ca^2+^ in mouse, pig, and human lungs. A 15-second hyperinflation due to airway pressure increase from 5 to 15 cmH_2_O induced prolonged Ca^2+^ increases (Figure 3, A, B, D, and F). The subsequent return of Ca^2+^ to baseline ruled out the possibility that the response was injury induced. Ca^2+^ increases did not occur when we increased airway pressure to a smaller extent, namely from 5 to 10 cmH_2_O (Supplemental Figure 3, A and B), indicating that the Ca^2+^ response was not sensitive to small increases of lung volume.

Although hyperinflation expanded all mouse alveoli, Ca^2+^ increases occurred in 35% of the AMs (Figure 3, C, E, and G). As exemplified, AMs that lacked Ca^2+^ responses were frequently next-alveolus neighbors of AMs that responded with Ca^2+^ increases (Figure 3B). We conclude, although hyperinflation caused epithelial stretch in all alveoli, the induced Ca^2+^ increases occurred in a subset of sessile AMs.

Human and pig alveoli were not edematous or injured, and they inflated well as delineated by epithelial fluorescence of calcein, which denoted cellular viability (Figure 1, B and C). Nevertheless, in these alveoli, cytosolic Ca^2+^ detection was not possible as acetoxymethyl-conjugated fluo-4 (fluo-4 AM) was not internalized by cells. In alveolar type 2 cells, mitochondrial buffering masks cytosolic Ca^2+^ increases due to lung hyperinflation (19). The buffering increases the mitochondrial Ca^2+^, which is therefore a surrogate for the cytosolic response. Since the mitochondrial Ca^2+^ indicator, rhod-2, was internalized by alveolar cells of human and pig lungs, we opted for mitochondrial Ca^2+^ detection in these lungs.

### Hyperinflation causes store release of Ca^2+^ in AMs.

**Figure 4:**
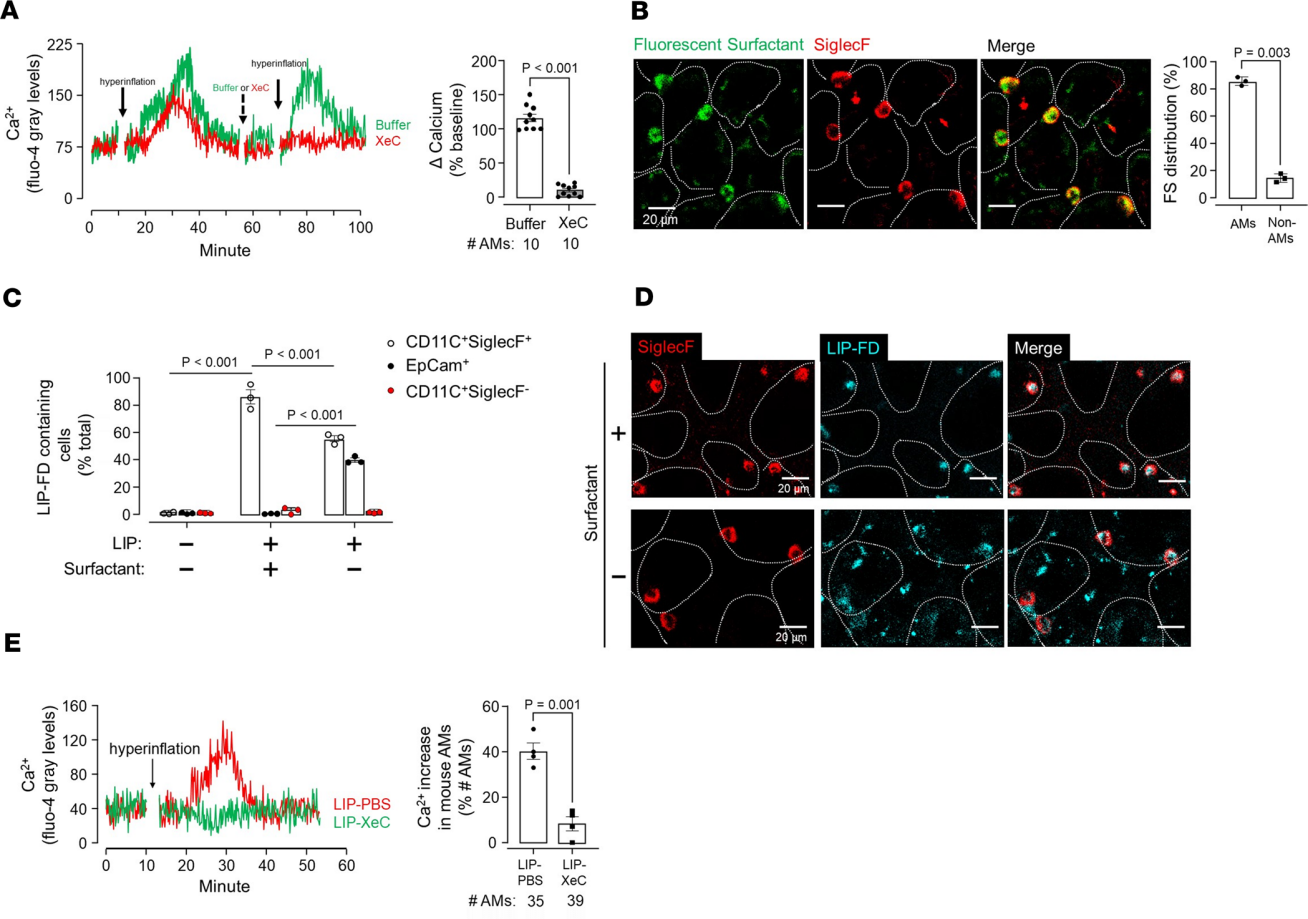
Targeted delivery of xestospongin C to AMs inhibits calcium mobilization. (**A**) Tracings in different colors show Ca^2+^ responses in different sessile AMs following sequential hyperinflation challenges as shown (solid arrows). Alveolar microinfusions of buffer alone (green tracing), or buffer containing xestospongin C (XeC) (red tracing) were given as shown (dashed arrow) in different lungs. Bars show the group data following the second hyperinflation. Findings were replicated in 10 AMs from 4 lungs. (**B**) Confocal images show uptake of fluorescent surfactant (FM1-43–labeled) in AMs 2 hours after intranasal instillation. Dotted lines trace alveolar perimeters. Bars show quantification of uptake in lungs. FS, fluorescent surfactant. (**C**) Bars show flow cytometry quantification of liposome uptake in AMs (CD11C^+^SiglecF^+^), dendritic cells (CD11C^+^SiglecF^–^), and alveolar epithelium (EpCam^+^) from lung cell suspension 2 hours after instillation. The liposomes contained rhodamine B–labeled dextran of molecular weight 70 kDa (LIP-FD). The gating strategy is shown in Supplemental Figure 3A and Supplemental Figure 9. *n* = 3 lungs each group. (**D**) Confocal images show AM (SiglecF^+^) uptake of LIP-FD 2 hours after intranasal instillation in presence (+, upper panel) or absence of surfactant (–, lower panel). Note, merge images show LIP-FD uptake in macrophages in the presence (upper right) but not absence (lower left) of surfactant. (**E**) Tracings show Ca^2+^ response in AMs of mice intranasally instilled with liposomes encapsulating PBS (LIP-PBS) or xestospongin C (LIP-XeC) 2 hours prior to lung imaging. Bars show quantification of the percentage of AMs responding with Ca^2+^ increase after hyperinflation in AMs of mice intranasally instilled with LIP-PBS or LIP-XeC 2 hours prior to lung imaging. Data analyses. Group data are mean ± SEM. *P* values were calculated by 1-way ANOVA with Bonferroni’s correction (**C**) or paired (**A**) or unpaired (**B** and **E**) *t* test.

To determine mechanisms underlying the hyperinflation-induced Ca^2+^ increase, we affirmed that when 2 hyperinflations were given consecutively, each caused a similar Ca^2+^ transient (Figure 4A). However, when given by alveolar microinfusion after the first hyperinflation, xestospongin C (XeC) inhibited the Ca^2+^ increase due to the second hyperinflation (Figure 4A). Since XeC blocks store release of Ca^2+^ (20), we interpret that hyperinflation-induced Ca^2+^ increases resulted from endosomal Ca^2+^ release.

Since alveolar microinfusion does not ensure cell-specific delivery, we developed a surfactant-based strategy (21) for XeC delivery to AMs. Intranasally instilled surfactant, which was fluorescently labeled with FM1-43, was engulfed by AMs (Figure 4B), indicating that the surfactant vehicle potentially provided a means for targeting liposomes (LIPs) to AMs. Hence, we prepared liposomes that encapsulated fluorescent dextran (LIP-FD), then gave the LIP-FD with surfactant. For control, we gave LIP-FD without surfactant.

Our flow cytometry findings indicated that surfactant-associated LIP-FD was predominantly internalized by AMs, which were identified as CD11c^+^SiglecF^+^ cells (Figure 4C, Supplemental Figure 4A, and Supplemental Figure 5). However, no LIP fluorescence was detected in CD11c^+^SiglecF^–^ cells. Since circulating monocytes, which are also SiglecF^–^, do not enter the alveolar spaces within 2 hours of injury induction (22), the CD11c^+^SiglecF^–^ cells are likely to be dendritic cells (DCs) (13) (Figure 4C and Supplemental Figure 5). LIP fluorescence was also absent in EpCam-positive cells, marking the alveolar epithelium (Figure 4C and Supplemental Figure 5). Hence, the alveolar epithelium and DCs did not internalize the liposomes. It is possible that about 10% of the uptake occurred in other cells, such as neutrophils. However, we did not detect LIP-FD uptake in spleen, heart, and liver (Supplemental Figure 4B), indicating that surfactant-associated LIP-FD were not taken up in systemic organs. By contrast, given without surfactant, LIP-FD were extensively taken up in the alveolar epithelium but not DCs, as detected by flow cytometry (Figure 4C and Supplemental Figure 5). These findings were affirmed by RCM data, which showed that surfactant-associated LIP-FD localized to AMs, while given without surfactant, LIP-FD were taken up by other cells (Figure 4D). Thus, inclusion of surfactant in the instillation was necessary to specifically target LIP delivery to AMs and, importantly, to avoid nonspecific delivery to other cell types.

The success of this macrophage-targeted delivery system motivated us to suspend LIPs containing XeC in surfactant (LIP-XeC). RCM studies in lungs of these mice indicated marked inhibition of hyperinflation-induced Ca^2+^ increase in sessile AMs (Figure 4E). By contrast, concomitant Ca^2+^ oscillations in the alveolar epithelium were not blocked (Supplemental Figure 6, A and B), indicating that the surfactant-associated delivery was AM but not epithelium targeted. Hence, the delivery of LIP-XeC had no effect on distension-induced epithelial Ca^2+^ responses. Taking these findings together, we conclude that the AM Ca^2+^ response to hyperinflation was initiated by Ca^2+^ release from endoplasmic stores.

### Cx43 determines Ca^2+^ mobilization in AMs.

In several experiments, as for in the example shown (Figure 3A), an AM pedicle was evident that appeared to anchor the AM on the epithelium. In these instances, the hyperinflation-induced Ca^2+^ increase occurred first in the pedicle before proceeding to the cell body of the AM (Figure 3A). These findings suggested that hyperinflation may have induced a Ca^2+^ wave that followed a trajectory from the pedicle to the cell body.

**Figure 5:**
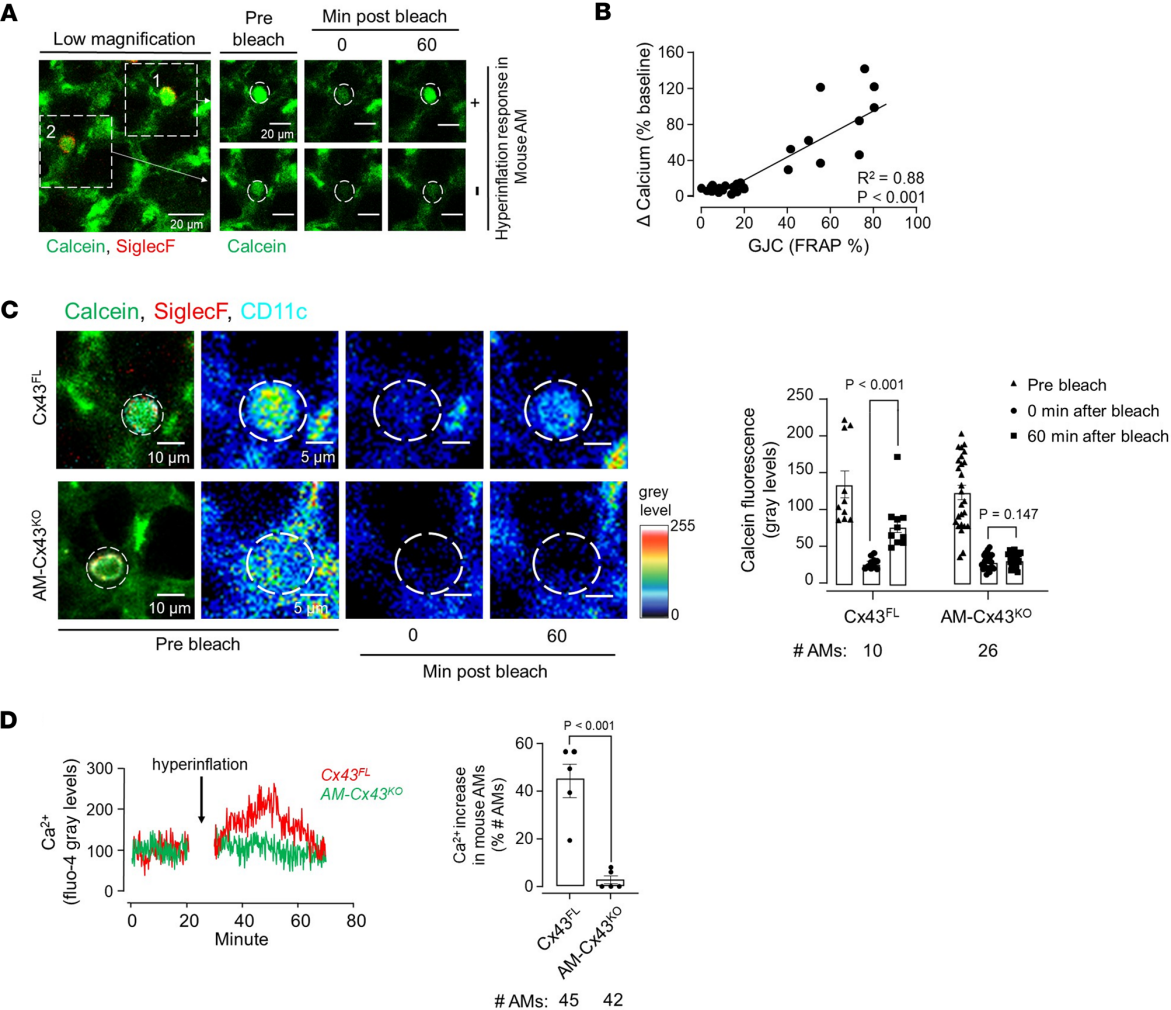
Role for connexin-43 in AM calcium mobilization. (**A**) Confocal image (left) of alveolar epithelium and SiglecF-positive AMs loaded with the cytosol-localizing dye, calcein. Dashed squares enclose 2 AMs subjected to photobleaching. High-magnification images show calcein fluorescence in a hyperinflation-responsive AM (#1, upper panel) and nonresponsive AM (#2, lower panel) before and after bleach at indicated time points. Replicated 8 times in 4 lungs. (**B**) Plot shows increase in cytosolic Ca^2+^ in AMs after hyperinflation against efficiency of gap junctional communication (GJC) quantified in terms of fluorescence recovery after photobleaching (FRAP). Data are for 27 AMs from 4 lungs. Line drawn by linear regression. (**C**) Confocal images show calcein green–loaded sessile AMs identified by SiglecF and CD11c labeling. Pseudocolor high-power images of sessile AMs are shown in prebleach and 0- and 60-minute after bleach periods. White dashed circles indicate photobleached regions. Bars show group data quantifications of calcein fluorescence within sessile AMs at indicated time points. *n* = 3 and 4 lungs, respectively, for Cx43^FL^ and AM-Cx43^KO^ groups. Cx43^FL^, floxed littermate control. AM-Cx43^KO^, CD11cCre-Cx43^fl/fl^. (**D**) Tracings in different colors show Ca^2+^ responses in AMs from indicated mice. Bars show percentage of AMs per field of view that responded to hyperinflation by increasing Ca^2+^ in CD11cCre-Cx43^fl/fl^ mice (AM-Cx43^KO^) and littermate controls (Cx43^FL^). Data analyses. Group data are mean ± SEM. *P* values were calculated by paired (**C**) or unpaired (**D**) *t* test.

Sessile AMs establish gap junctional communication (GJC) with the alveolar epithelium through Cx43-containing gap junctions (13). Hence, we assessed efficacy of the GJC by quantifying fluorescence recovery after photobleaching (FRAP) (13). The GJC efficacy varied between AMs, as exemplified by the images from single experiments (Figure 5A). The plot shows that the hyperinflation-induced Ca^2+^ response correlated positively with the GJC efficacy (Figure 5B). In fact, when FRAP was less than 20% of the baseline fluorescence, the Ca^2+^ increase was negligible. In transgenic mice lacking Cx43 in sessile AMs (13) (AM-Cx43^KO^), which therefore lacked GJC (Figure 5C), the images and the group data show hyperinflation-induced Ca^2+^ increases were absent as compared with floxed littermate controls (Cx43^FL^) (Figure 5D). Together, these findings indicated that AM-epithelium communication by Cx43-dependent GJC sufficiently accounted for the hyperinflation-induced Ca^2+^ increases in sessile AMs, further ruling out macrophage stretch as the underlying mechanism.

### Mechanosensitive TNF-α secretion occurs in AMs.

A transient Ca^2+^ increase induces vesicular exocytosis, hence secretion (23). Hence, we next asked whether the hyperinflation-induced Ca^2+^ increase in sessile AMs was of sufficient duration and magnitude as to activate secretion of TNF-α, the critical initiator of pro-inflammatory responses. In macrophages transfected in vitro with a plasmid expressing GFP-TNF-α (24), LPS or cytokine exposure initiates TNF-α secretion with a delay of hours, during which TNF-α–containing vesicles dock on the cell membrane (24). Although hyperinflation increases TNF-α levels in the bronchoalveolar lavage (BAL) (25, 26), it is not clear whether AM interactions with neighboring cells determine the secretion.

**Figure 6:**
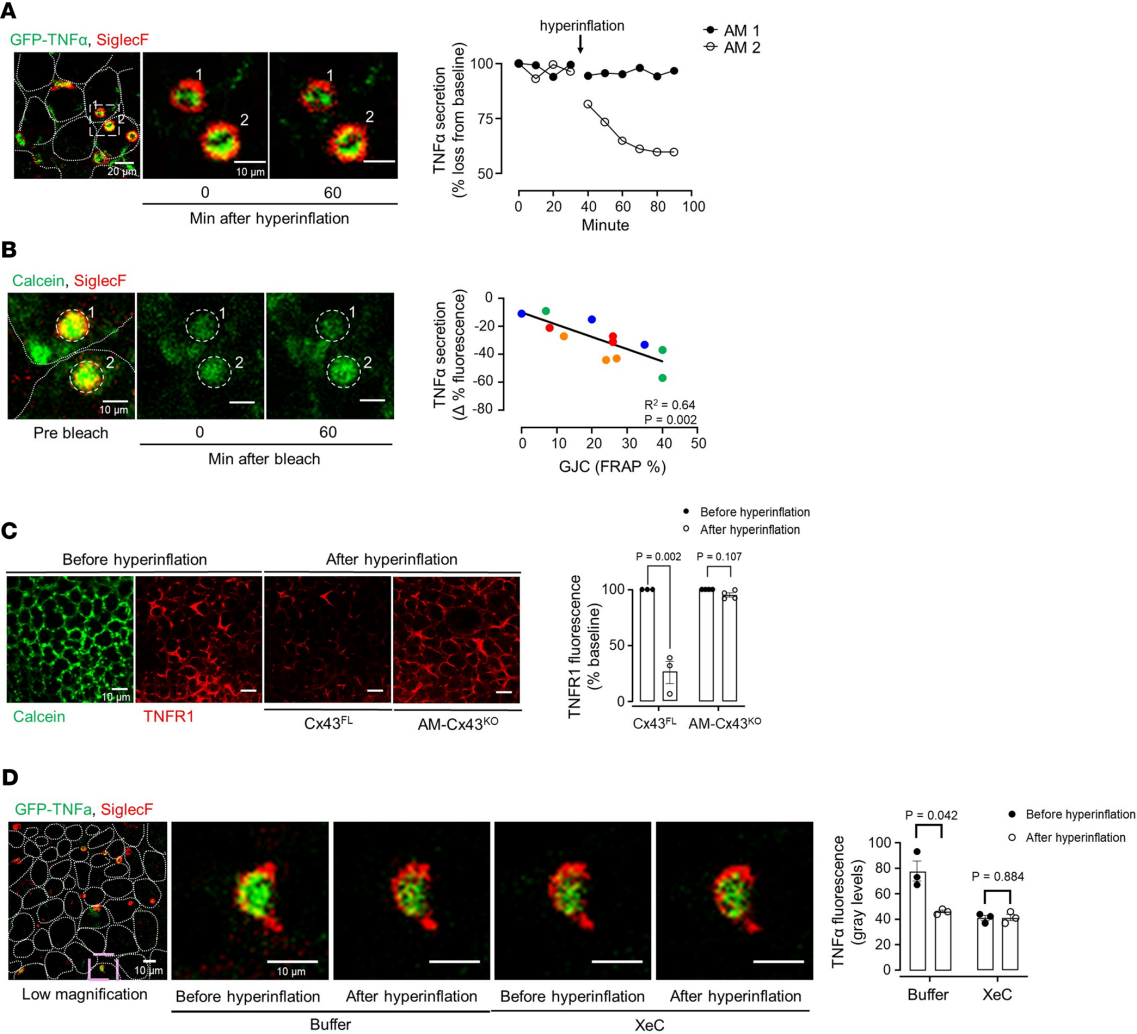
Hyperinflation induces TNF-α secretion in sessile AMs. (**A**) Confocal image (left) shows overlay of the indicated fluorescence labels on sessile AMs. Dotted lines indicate alveoli in which sessile AMs are located. Selected AMs (rectangle), imaged at high magnification (middle and right images), show TNF-α fluorescence before and after hyperinflation. The tracings depict fluorescence quantification at the indicated times. Findings were replicated in at least 3 AMs each in 3 lungs. (**B**) Confocal image (left) shows overlay of the indicated fluorescence labels on sessile AMs from same imaging field as **A**. Dotted lines indicate alveolar epithelial margins. Confocal images (middle and right images), show calcein fluorescence alone after laser bleach at indicated times. Dashed circles indicate laser bleached regions. Regression plot shows data for individual AMs from 3 lungs. Same color indicates AMs from an individual lung. TNF-α secretion is quantified as percentage fluorescence decrease from baseline. Line drawn by linear regression analysis. GJC, gap junction communication; FRAP %, fluorescence recovery after photobleaching expressed as difference from initial fluorescence, quantified 60 minutes after bleaching. (**C**) Confocal images of epithelial and TNFR1 fluorescence in alveoli microinfused with calcein (green) and anti-TNFR1 mAb (red) in littermate control mice (Cx43^FL^) and in mice with AM-specific Cx43 knockout (AM-Cx43^KO^) as indicated. Bars show TNFR1 shedding response. (**D**) Confocal image (left) shows overlay of the indicated fluorescence labels on sessile AMs. Selected AM (purple rectangle in the left image), imaged at high magnification, shows TNF-α fluorescence before and after hyperinflation in the absence (Buffer) or presence of XeC. Bars show quantification of TNF-α secretion. Data analyses. Group data are mean ± SEM. *P* values were calculated by paired (**C** and **D**) *t* test.

To evaluate this question, we intranasally instilled LIPs encapsulating GFP-TNF-α plasmid in surfactant to transfect sessile AMs in vivo. After 48 hours, a majority of sessile AMs expressed TNF-α fluorescence with a polar distribution in that the fluorescence was dominantly expressed at the lumen-facing cytosol (Figure 6A and Supplemental Figure 7). Hyperinflation rapidly decreased TNF-α fluorescence (Figure 6A), indicating that the cytokine was secreted. However, the secretion response was absent in several AMs. The extent of secretion was higher for AMs that had higher GJC efficacy (Figure 6B). Further, shedding of alveolar TNF-α receptor 1 (TNFR1), a marker of TNF-α ligation (27), was blocked in AM-Cx43^KO^ mice (Figure 6C). To determine the role of AM Ca^2+^ in the secretion response, we gave alveolar microinfusion of buffer or XeC prior to hyperinflation. XeC but not buffer blocked hyperinflation-induced TNF-α secretion in AMs (Figure 6D), indicating that the secretory response resulted from Ca^2+^ release from endoplasmic stores.

Taking our findings together, we interpret that inhibition of GJC in the knockout mice blocked the Ca^2+^ increase, hence release of TNF-α that ligated TNFR1. Thus, GJC between AMs and the epithelium was critical for the hyperinflation-induced TNF-α secretion.

### Lung injury induced by high tidal volume ventilation results from Cx43-dependent Ca^2+^ increases in AMs.

We next considered whether preexisting lung injury modifies pro-inflammatory responses initiated by the hyperinflation-induced, Cx43-dependent Ca^2+^ increases. Hence, we mechanically ventilated mice at high tidal volume (HTV), which causes lung inflammation and alveolar epithelial injury (28). Our goal was to match the lung hyperinflation due to HTV with that for the single hyperinflation applied in the RCM studies. Hence, we mechanically ventilated mice at HTV (18 mL/kg) or low tidal volume (LTV) (6 mL/kg). The end-inspiration airway pressure for HTV was higher than LTV by about 10 cmH_2_O, similar to the pressure increase we induced in the RCM studies. Further, since mouse total lung capacity (TLC) is approximately 1 mL (29), the HTV (~540 μL), added to mouse functional residual capacity of ~250 μL (29), accounts for lung expansion to about 80% TLC in a 30 g mouse. Thus, the lung expansion in HTV matched that for the single hyperinflation studies.

**Figure 7:**
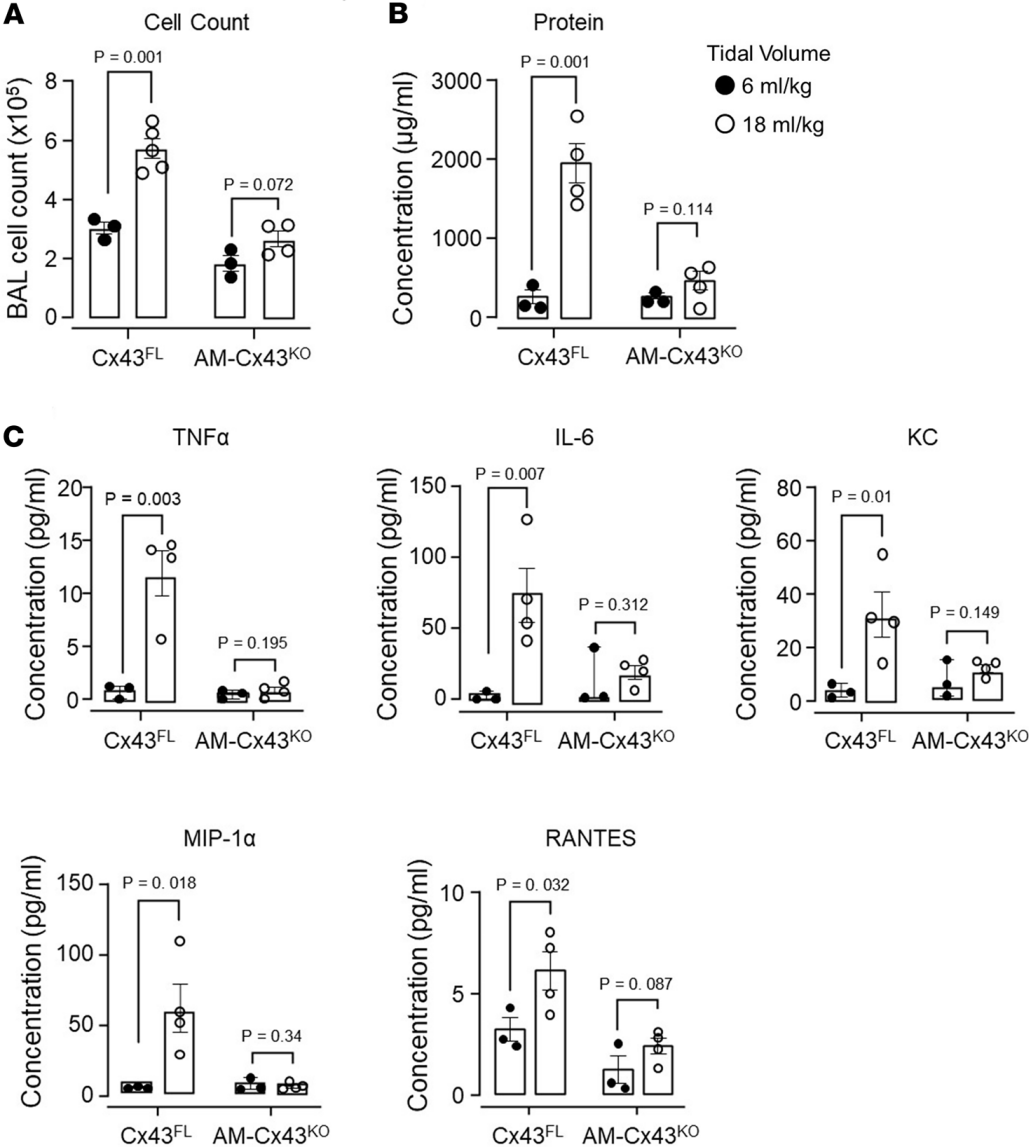
Sessile AM connexin-43 deletion protects against high tidal volume ventilation-induced lung injury. (**A**–**C**) Bars show quantifications in the bronchoalveolar lavage (BAL) for total cells (**A**), protein concentration (**B**), and cytokine and chemokine levels (**C**) in mice 2 hours after mechanical ventilation at indicated tidal volumes. Data analyses. Group data are mean ± SEM. Each dot represents data for 1 lung. *P* values were calculated by unpaired *t* test.

In control Cx43^FL^ mice, 2 hours of HTV induced major lung inflammation as indicated by increased content of total cells in the BAL (Figure 7A). We quantified alveolar barrier permeability in terms of the BAL protein content, which increased more than 2 times (Figure 7B). BAL levels of the pro-inflammatory factors, TNF-α, IL-6, keratinocyte chemoattractant (KC), macrophage inflammatory protein-1α (MIP-1α /CCL3), and RANTES (CCL5), increased 2- to 3-fold above baseline (Figure 7C). These injury responses were markedly inhibited in AM-Cx43^KO^ mice. We conclude, Cx43 expression in AMs critically determined HTV-induced lung inflammation and injury.

**Figure 8:**
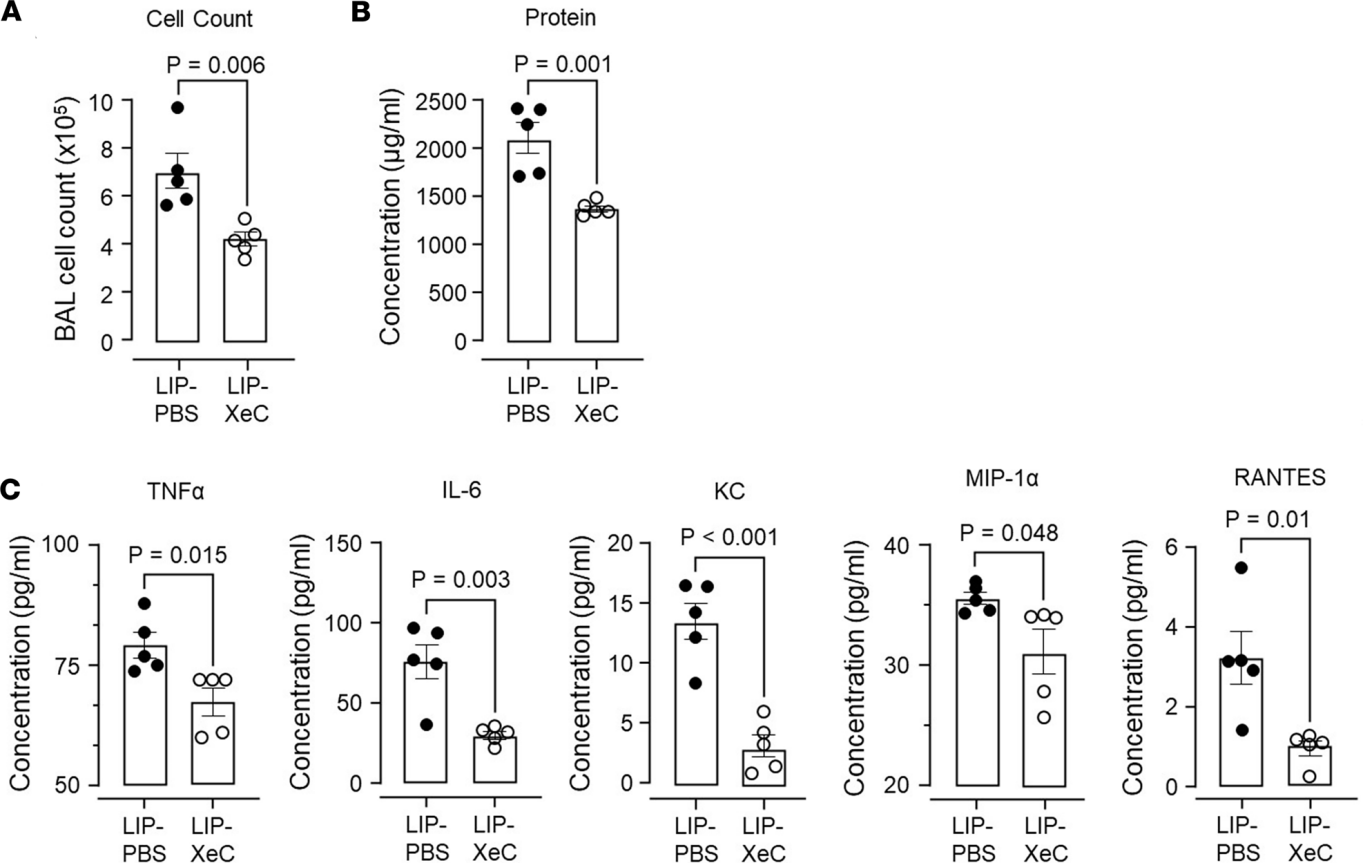
LIP-XeC given therapeutically attenuate high tidal volume ventilation-induced lung injury. (**A**–**C**) Bars show quantifications in the BAL for total cells (**A**), protein concentration (**B**), and cytokine and chemokine levels (**C**) in mice mechanically ventilated for 1 hour at 18 mL/kg then instilled with LIPs encapsulating XeC or PBS in a surfactant solution. Then ventilation continued for 1 hour. LIPs were given in a surfactant-containing solution. Data analyses. Group data are mean ± SEM. Each dot represents data for 1 lung. *P* values were calculated by unpaired *t* test.

Cre recombinase driven by the CD11c promoter deletes Cx43 in AMs and DCs. To rule out a role of DCs in the present responses, we relied on our finding that intranasal instillation of LIPs in surfactant specifically targets AMs. Since AMs face the airway lumen, they are amenable to this targeting, as different from DCs that lie on the interstitial aspect of the airway epithelium that forms a barrier to passive transepithelial fluid flows. Thus, as we reported above, instilled with surfactant, LIP-XeC was predominantly taken up by AMs (Figure 4, C and D), where it blocked Ca^2+^ increases (Figure 3E), indicating that the instillation reached AMs and not other cells, including DCs. To determine effects on the HTV response, we established HTV for 1 hour. Then, we instilled LIP-XeC in surfactant, and we continued HTV for another hour. Subsequent BAL analyses indicated that LIP-XeC instillation decreased the HTV-induced BAL markers of lung inflammation and injury (Figure 8, A–C). LIPs that encapsulated PBS were not protective. These findings indicate that AM-targeted inhibition of Ca^2+^ increases by XeC ameliorated HTV-induced lung injury.

## Discussion

**Figure 9:**
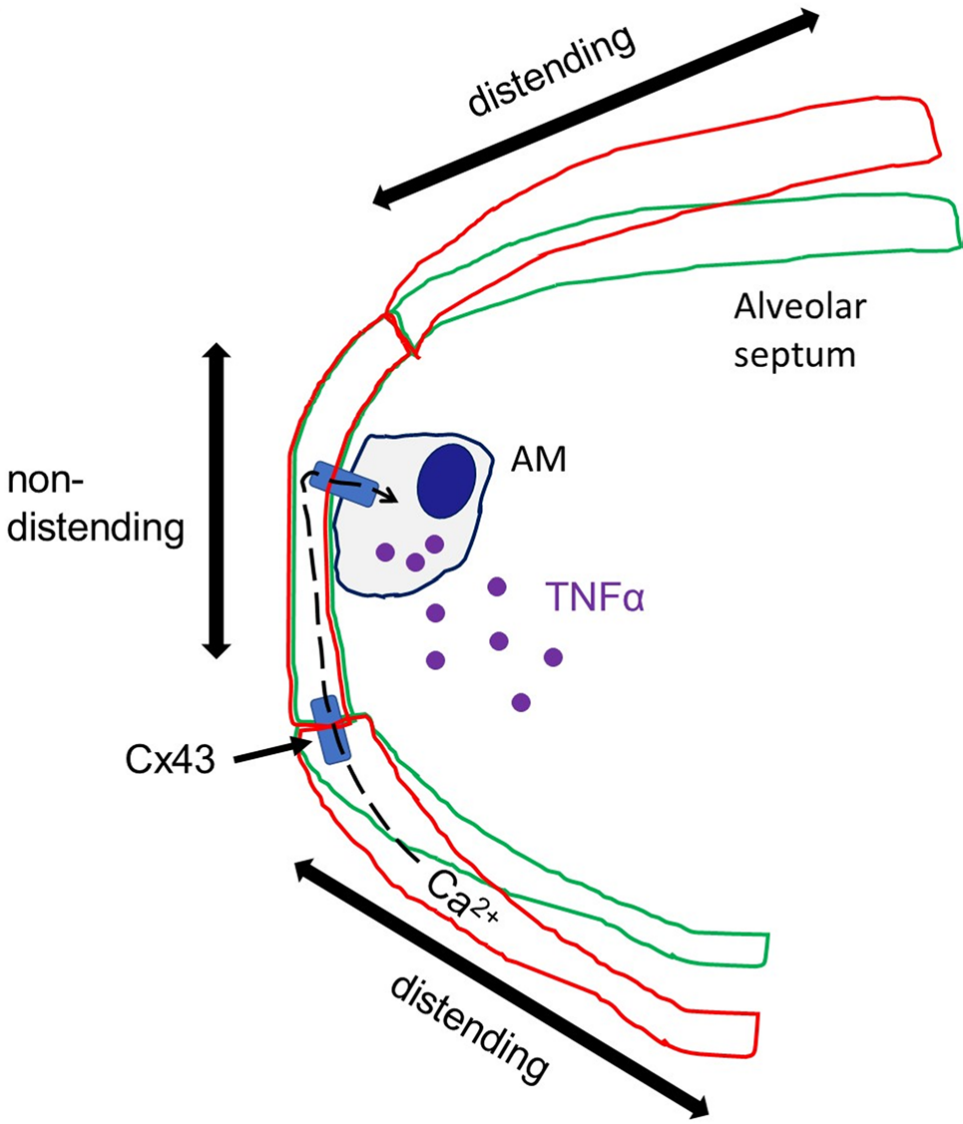
Diagrammatic depiction of alveolar wall responses underlying hyperinflation-induced mechano-immunity. The sketch depicts an AM in the alveolar corner formed by the curvature of the alveolar wall that lies between flat alveolar septa. Alveolar expansion from baseline (green) to hyperinflation (red) distends AM-free alveolar septa but not the AM-containing alveolar corner. Ca^2+^ increase in the distended segment diffuses intercellularly through Cx43 gap junctions to AMs in the nondistended segment. The Ca^2+^ increase in AMs initiates TNF-α secretion.

Our goal was to test the prevailing view that stretch-induced AM activation underlies the lung’s mechanosensitive immune response (5). We show here that although alveolar expansion caused no dimensional changes in AMs, lung immune responses were induced in terms of AM activation and the inflammatory response in the BAL. To determine whether nonuniformity of alveolar strain was a factor in these responses (8), we interrogated the pattern of hyperinflation-induced shape changes in alveoli that contained AMs. These studies revealed that while flat septa were strain sensitive, AMs were located at strain-protected regions at alveolar curvatures formed between flat septa (Figure 9). Thus, AM-containing alveolar segments did not distend, so the AMs did not stretch. In previous studies, we noted that alveolar liquid (30) and inhaled bacteria (31) tend to accumulate at these acute curvatures. Mechanisms that determine such site-specific accumulations in alveoli, including that of AMs as per present data, require further understanding. Regardless, our findings definitively rule out imposed macrophage stretch as the critical mechanism that underlies the lung’s immune response to hyperinflation.

These interpretations must be made with caution since our RCM findings were obtained in subpleural alveoli that may expand differently from alveoli in deeper lung regions. However, we note that although regional variation of lung expansion depends on variation in the transpulmonary pressure, alveoli themselves expand similarly at subpleural and deeper lung levels as reported in classical studies (32). Given this similarity, we anticipate that strain heterogeneities and AM location in strain-protected alveolar segments occurs in all alveoli. The mechanistic basis for these strain differences remains unclear, but an issue worth exploring is whether they contribute to the overall stability of alveolar expansion.

The puzzle of how the Ca^2+^ increases occurred despite lack of AM stretch was resolved through FRAP assays that revealed GJC between AMs and the adjacent alveolar epithelium. Inhibition of the GJC by *Cx43* deletion in AMs blocked the hyperinflation-induced responses in AMs. Further, AM-targeted LIP-XeC delivery blocked both Ca^2+^ increase and TNF-α secretion in AMs. Taking these findings together, we conclude that hyperinflation activated Ca^2+^ communication between the alveolar epithelium and AMs across Cx43-dependent gap junctional channels, resulting in TNF-α secretion. Hence, the presence of Cx43 in AMs, but not AM stretch, critically determined the lung’s mechanosensitive immune response. Approximately a third of all AMs tested in mouse, human, and pig lungs responded with Ca^2+^ increases to hyperinflation. The similarity of these responses suggests translational relevance of the mouse findings. However, this interpretation needs to be made with caution, and further studies need to be designed to improve understanding of whether prolonged periods of HTV progressively induces Ca^2+^ responses in AMs that are initially nonresponsive.

The importance of these mechanisms as determinants of the lung’s global mechano-immune response was revealed by mechanically ventilating mice at HTV. These mice developed marked increases in the neutrophil count as well as in the levels of pro-inflammatory cytokines and plasma proteins in the BAL, affirming reported findings (33). However, AM-specific *Cx43* deletion blocked all these responses. Thus, our findings in mechanically ventilated mice were similar to those in isolated lungs, in that both models Cx43-containing GJC was revealed as the critical injury-initiating mechanism. We speculate that Ca^2+^-induced TNF-α secretion from AMs followed by ligation of TNFR1 on the alveolar epithelium induced a sequence of events that led to secretion of multiple cytokines, causing lung injury as reflected by increased barrier permeability to plasma proteins.

The burgeoning interest in AM-specific drug delivery addresses the critical role played by AMs in initiating and sustaining lung injury (34). Surfactant given alone is ineffective in improving mortality in mechanically ventilated patients with ARDS (35). However, we show here that surfactant might be an effective vehicle for AM therapy as it mediated AM-specific delivery of LIPs. Notably, surfactant-associated LIPs were not taken up in the alveolar epithelium, or in systemic organs, ruling out the possibility of potential nonspecific effects. By contrast, given without surfactant, LIPs were taken up nonspecifically as we show here and others report (36). Mannosylated albumin nanoparticles have been directed at CD206-expressing AM receptors (37). However, since all AMs internalize surfactant (38), by present strategy, surfactant-associated LIPs are likely to target all AMs. Accordingly, the instillation of surfactant-associated LIP-XeC blocked the hyperinflation-induced Ca^2+^ increase in AMs, as well as the lung injury response to HTV. A role for DC targeting in this protective response may be ruled out, as we did not detect substantial uptake of LIPs in DCs. To our knowledge, our findings indicate airway instillation of surfactant-associated LIPs provides what we believe to be a novel therapeutic strategy for inhibiting lung injury through AM-specific targeting.

An important set of findings relates to the comparison of responses in mouse, human, and pig alveoli. Although such comparisons are likely to advance translational understanding, a caveat is that human lungs are transplant rejects and are therefore not pristine, potentially complicating comparisons against findings in rodent models. We addressed these difficulties through studies in pig alveoli, since pig lungs are pristine, and they provide a translational model for studies of mechanical ventilation (10, 11). The failure of hyperinflation to cause AM shape changes in mouse and pig alveoli, as also in human alveoli, suggests that our interpretation that AMs are strain protected is translationally valid.

The increase of AM Ca^2+^ emerged as the main hyperinflation-induced immunogenic mechanism. Although these mechanisms need to be better understood, we suggest that immune suppression versus activation might depend on the nature of the Ca^2+^ waves that traverse gap junctional channels between AMs and the alveolar epithelium. Our reported data indicate that airway-instilled LPS activates immunosuppressive episodic Ca^2+^ waves that travel from the AMs to the epithelium, occurring every 10–15 minutes (13). Here, by contrast, hyperinflation-induced epithelial stretch induced immunogenic Ca^2+^ waves in the opposite direction, namely from epithelium to the AM. This stretch-induced Ca^2+^ wave was prolonged, lasting up to 20 minutes, and they occurred every time the epithelium was stretched. Thus, the profiles of immunosuppressive versus immunogenic Ca^2+^ waves are distinctly different. These AM-induced, Ca^2+^-determined immune mechanisms require further study.

Our findings extend micromechanical understanding of the complex strain landscape of the distended alveolar wall. What is now clear in a translationally relevant manner is that the nondistending segment of the alveolus forms the key protective niche for AMs, obviating the need to posit AM stretch as the critical mechanism underlying immune activation in mechanical ventilation. Nevertheless, the AMs formed the hub for the pro-inflammatory signaling responsible for tissue injury during ventilatory lung overexpansion, since pro-inflammatory Ca^2+^ signals passed from the epithelium to the AMs across gap junctions. We point out, however, our study did not include consideration of prolonged durations of ALI that might modify these mechanisms. For example, injury that persists for days or weeks may involve responses in not only resident AMs but also newly recruited, monocyte-derived AMs. The extent to which resident and recruited AMs continue to form Cx43 bridges with the epithelium and thereby impact inflation-induced lung injury requires further study. Despite these shortcomings, we suggest that inhibition of the Cx43-dependent Ca^2+^ signaling in AMs might lead to new therapy for AM-initiated inflammatory lung diseases.

## Methods

### Sex as a biological variable.

Sex was not considered as a biological variable in the present studies.

### Mice.

All mice were cared for according to the NIH *Guide for the Care and Use of Laboratory Animals* (National Academies Press, 2011). Mice were socially housed under a 12-hour light/12-hour dark cycle with ad libitum access to water and food. The mice were inbred, so no randomization was required. Nevertheless, we randomly assigned age-matched mice to experimental groups. Mice were between 2 and 4 months and on a C57BL/6J background. CD11cCre^+/–^ mice were provided by Boris Reizis (Columbia University) while Cx43^fl/fl^ (stock no. 008039) and C57BL/6J (stock no. 000664) mice were purchased from the Jackson Laboratory and bred in-house.

### Materials.

A list of antibodies used in this study is available in Supplemental Table 1. We purchased mAb MCA2350 against the TNFR1 extracellular epitope (40 μg/mL) from AbD Serotec. Anti-TNFR1 antibody was fluorescently labeled with Alexa Fluor 633 using our standard protocol (39). Fluo-4 AM (5 μM), Lysotracker Red (LTR, 100 nM), calcein AM (5 μM), FM1-43 (5 μM), and rhodamine-B–labeled dextran 70 kDa (80 μg) were purchased from Thermo Fisher Scientific. XeC (20 μM) was purchased from Sigma-Aldrich. We purchased human recombinant TNF-α (10 ng/mL) from BD Biosciences. Surfactant (CUROSURF, poractant alfa, Chiesi Farmaceutici) was used at a final concentration of 5 mg/mL. FM1-43 dye (5 μM) and surfactant (5 mg/mL) were incubated together away from light for 10 minutes at room temperature to generate fluorescent (FM1-43–labeled) surfactant. Vehicle for fluorophores, antibodies, and other agents was HEPES buffer (see below for composition).

### Isolated, blood perfused mouse lung preparation.

Using our reported methods (19), we anesthetized (intraperitoneal [I.P.], ketamine 100 mg/kg, xylazine 10 mg/kg) and heparinized mice (1,000 IU/kg, intracardiac). Then we exsanguinated mice and excised the lungs for pump perfusion (0.5 mL/min, 37°C) through the pulmonary artery (PA) using autologous blood (1 mL of 5 mL total perfusate) with added HEPES buffer (150 mmol/L Na^+^, 5 mmol/L K^+^, 1.0 mmol/L Ca^2+^, 1 mmol/L; Mg^2+^, and 20 mmol/L HEPES, pH 7.4) containing 70 kDa dextran (4% w/v) and 1% FBS. Osmolarity was 300 mosM (Fiske Micro-Osmometer, Fiske Associates). The left atrial outflow was recirculated through the PA. Lungs were inflated with room air through a tracheal cannula. We held the PA, left atrial, and airway pressures at 10, 3, and 5 cmH_2_O, respectively, during microscopy (Supplemental Figure 8).

### Alveolar micropuncture and microinfusion.

Using glass micropipettes (tip diameter 3–5 μm), we microinfused single alveoli with fluorescent dyes and antibodies (Supplemental Figure 8). Infusates were delivered to ~7 alveoli around the micropuncture site (19). For each bolus, we microinfused alveoli for ~3 seconds after which the liquid drained from the alveolar lumen in seconds, reestablishing air-filled alveoli. This rapid clearance indicates that the micropuncture did not rupture the alveolar wall and that the micropunctured membrane rapidly resealed as reported for other cells (40). Nevertheless, we selected non-micropunctured alveoli for imaging. To determine whether the fluorescence detected in the epithelium was intracellular or extracellular, in some experiments we microinfused alveoli with the fluorescence quencher, trypan blue (0.01% w/v, Thermo Fisher Scientific). Persistent fluorescence after trypan blue washout indicated intracellular labeling (39). In all experiments in which multiple dyes were infused, we confirmed absence of bleed-through between fluorescence emission channels.

### Liposome preparation.

Following the manufacturer’s protocol, we extruded unilamellar LIPs (20 μg/μL; 100 nm pore size; DOTAP, Avanti Lipids) in sterile Opti-MEM (Invitrogen). We used freshly extruded LIPs, then incorporated in them plasmid DNA, rhodamine-labeled dextran 70 kDa, XeC, or PBS as required, per our established protocol (39).

### In vivo mouse sessile AM lung transfection.

GFP-TNF-α plasmid (41) (plasmid #28089) was purchased from Addgene. Using our established methods (19), we incorporated plasmid DNA (80 μg) in LIPs (480 μg lipid content) in sterile Opti-MEM. We administered the LIPs (60 μL) in a surfactant-containing suspension (5 mg/mL) by intranasal (I.N.) instillation in anesthetized mice. Imaging experiments were carried out 48 hours after transfection.

### Live imaging of mouse lungs.

We imaged intact alveoli in a 1.7 μm–thick optical section (512 × 512 pixels) at a focal plane ~20 μm deep to the pleural surface of live lungs with laser scanning microscopy (TCS SP8, Leica Microsystems) using a 10× air objective (numerical aperture 0.3, Leica Microsystems) or 25× water immersion objective (numerical aperture 0.95, Leica Microsystems) (Supplemental Figure 8). For *z* stacks, we imaged 1.7 μm–thick optical sections (512 × 512 pixels) at vertical intervals of 1.7 μm from the pleural surface to a depth of 40 μm. To immerse the 25× objective in water, we placed a waterdrop on a coverslip that was held in a metal O-ring, as described previously (19). We used our reported methods to detect fluorescence of microinfused dyes and antibodies in live alveoli (13). Cytosolic Ca^2+^ was detected using the cytosolic Ca^2+^ indicator, fluo-4 AM, an acetoxymethyl ester that fluoresces upon intracellular cleavage of the acetoxymethyl moiety as previously reported (13). Mitochondrial Ca^2+^ was detected using the mitochondrial Ca^2+^ indicator, rhod-2-acetoxymethyl, as previously reported (19). We recorded Ca^2+^ responses in alveolar epithelium and macrophages at 1 image/10 seconds as previously reported (13). All images were recorded as single images and processed using ImageJ (v15.3, NIH). We applied brightness and contrast adjustments to individual pseudocolor channels of entire images and equally to all experimental groups. No further downstream processing or averaging was applied.

### Mouse lung hyperinflation.

To induce lung hyperinflation, we increased airway pressure from 5 to 15 cmH_2_O for 15 seconds. After 15 seconds we reduced airway pressure to 5 cmH_2_O and identified the prehyperinflation optical section using morphological landmarks as previously described (8). To confirm alveolar stretch due to hyperinflation in mouse lungs, we recorded the time-dependent secretion of surfactant by alveolar epithelial type II cells as loss of LTR fluorescence after hyperinflation as shown (Supplemental Figure 9) and as previously described (42).

### Live imaging of pig lungs.

Confocal microscopy on live pig lungs was carried out in lungs obtained from 2 male and 1 female (3-month-old) Yorkshire strain pig donors. Pigs were anesthetized by intramuscular injection of ketamine (100 mg/kg) and xylazine (10 mg/kg). The lungs were removed immediately after the pig was euthanized by potassium chloride overdose by intracardiac injection. The lingular lobe, which provides a flat surface convenient for live microscopy, was positioned below the objective of a 2-photon microscope (TCS SP8). The lobe’s PA pressure was held at 10 cmH_2_O, while the lobe was inflated at alveolar pressure of 5 cmH_2_O through a bronchial cannula. We injected fluorescent dyes and antibodies through a 31-gauge needle into alveoli. To induce alveolar expansion, lungs were inflated by increasing airway pressure from 5 to 15 cmH_2_O. For a single hyperinflation protocol, airway pressure was increased from 5 to 15 cmH_2_O for 15 seconds.

### Live imaging of human lungs.

Confocal microscopy on live human lungs was carried out in lungs obtained from 3 male and 1 female (ages 34–82) deidentified donors. The lungs were imaged after about 20 hours of cold ischemia. Human lungs were warmed with perfusion of 37°C buffer prior to and during imaging. The temperature of the lung’s venous outflow was directly measured and set at 37°C, which represented the lung’s core temperature. The lingular lobe, which provides a flat surface convenient for live microscopy, was positioned below the objective of a 2-photon microscope (TCS SP8). The lobe’s PA pressure was held at 10 cmH_2_O, while the lobe was inflated at alveolar pressure of 5 cmH_2_O through a bronchial cannula. We injected fluorescent dyes and antibodies through a 31-gauge needle into alveoli. To induce alveolar expansion, lungs were inflated by increasing airway pressure from 5 to 15 cmH_2_O. For a single hyperinflation protocol, airway pressure was increased from 5 to 15 cmH_2_O for 15 seconds. Human lungs were rejected for the experiment if: (a) there was presence of exudates and cells denoting alveolar disease and (b) there was a failure to detect alveolar expansion following a hyperinflation pressure challenge.

### FRAP.

In RCM studies of live mouse lungs, we quantified FRAP, which assays GJC as per our previous protocol (13, 43). We first identified sessile AMs (SiglecF^+^ cells) in which the Ca^2+^ or TNF-α secretory response was present or absent, and then we determined GJC by FRAP. We photobleached cytosolic fluorescence within AMs and determined fluorescence recovery indicating intercellular dye transfer and GJC with alveolar epithelium. Lack of fluorescence recovery indicates lack of GJC.

### I.N. and intratracheal instillation.

For I.N. instillation, mice were anesthetized (I.P., ketamine 50 mg/kg, xylazine 5 mg/kg), then instilled with 2 mL/kg of instillate and allowed to recover for 2 hours prior to the experiment. For intratracheal instillation during MV protocol, ventilation was paused by removing the tracheal cannula from the ventilator, and then anesthetized mice were instilled with 2 mL/kg of instillate through the cannula. The cannula was reconnected to the ventilator after instillation. For LIP-XeC instillation, mice were I.N. instilled with 20 μM XeC complexed in LIPs (20 μg/μL) suspended in a surfactant-PBS (Ca^2+^ and Mg^2+^ free) suspension (5 mg/mL). A total of 60 μL of LIP-XeC suspension was instilled.

### MV.

A previously described MV protocol with modifications was used (28). Briefly, mice were anesthetized with isoflurane (4%) and ketamine (50 mg/kg) and xylazine (5 mg/kg) administered I.P. A tracheal cannula (PE-90 tubing, BD Biosciences) was inserted and secured into place by suture, and MV was started with a tidal volume of 6 mL/kg, a positive end-expiratory pressure of 5 cmH_2_O, and a respiratory rate of 120 breaths/min (VentElite, Harvard Apparatus). Following a 10-minute stable baseline period, in some experiments tidal volume was incrementally increased to 18 mL/kg over 5 minutes for HTV ventilation or continued at 6 mL/kg for LTV ventilation. Depth of anesthesia was assessed by reaction to paw pinch throughout the protocol. Anesthesia was maintained with ketamine (20 mg/kg) and xylazine (2 mg/kg) both administered by I.P injection. MV was continued for 2 hours. Saline (2 mL/kg) was administered in 30-minute intervals by I.P. injection throughout the protocol. Blood oxygen saturation, airway pressures, blood pressure, and heart rate were monitored continuously using a computer-integrated data collection system (MouseOx Plus, Starr Life Sciences). Temperature was monitored using a rectal probe, and temperature was maintained at 37°C throughout the protocol using a heating blanket (Homeothermic Blanket Control Unit, Harvard Apparatus). After 2 hours of MV, BAL was performed for analysis of BAL fluid and ALI was evaluated.

### Analysis of BAL fluid.

The lungs of mice were lavaged repeatedly (5 times) with the same 1 mL ice-cold PBS (Ca^2+^ and Mg^2+^ free) through a tracheal cannula. Collected BAL fluid was centrifuged for 4 minutes at 400*g* at 4°C. The supernatant was analyzed for protein and cytokine concentrations. We measured TNF-α, IL-6, KC (CXCL1), MIP-1α (CCL3), and RANTES (CCL5) concentrations in BAL fluid to determine pro-inflammatory cytokine secretion. Sample testing was carried out by Quansys Biosciences using a multiplex chemiluminescence assay (Q-plex) for the detection of mouse cytokines.

### Evaluation of ALI.

We evaluated ALI by assessing BAL protein content by BCA assay (Thermo Fisher Scientific) to determine protein leak from vascular compartment into alveolar airspace. We also assessed ALI by characterizing cell populations in BAL by flow cytometry (see below).

### Flow cytometry analysis of BAL cells.

Briefly, BAL cell pellet was resuspended in PBS (Ca^2+^ and Mg^2+^ free) supplemented with 1% FBS and 100 mM HEPES and used for flow cytometry studies. Relevant antibodies were added to the cell suspension and incubated away from direct light for 1 hour at room temperature. The cells were centrifuged (4 minutes, 4°C, 400*g*) and resuspended in buffer. This procedure was repeated 2 more times, and the cells were finally resuspended in 300 μL PBS (Ca^2+^ and Mg^2+^ free) without supplements. We analyzed cells by flow cytometry (3L Cytek Aurora, Becton Dickinson) according to manufacturer’s protocols using standard software (FCS Express 7 Flow, De Novo Software).

### Flow cytometry analysis of LIP uptake by lung cells.

Mice were I.N. instilled with 80 μg rhodamine-B–labeled dextran 70 kDa (Thermo Fisher Scientific) complexed in LIPs (480 μg total) suspended in a surfactant-containing suspension (5 mg/mL). A total of 60 μL of LIP-surfactant suspension was instilled. Then, 2 hours later, we isolated lung cells. To isolate cells, we buffer (PBS, Ca^2+^, and Mg^2+^ free) perfused lungs through vascular cannulas to clear blood; we then minced and passed the tissue through 40 μm cell strainers (BD Biosciences) to obtain a single-cell suspension. For flow cytometry, we surface-stained the cells by incubating the suspension with relevant antibodies away from direct light for 1 hour at room temperature. We analyzed cells by flow cytometry (5L Cytek Aurora, Becton Dickinson) according to manufacturer’s protocols using standard software (FCS Express 7 Flow, De Novo Software). AMs were identified as SiglecF-PE– and CD11c-APC–positive cells following a standard gating protocol (Supplemental Figure 5) (44). DCs were identified as SiglecF-PE–negative and CD11c-APC–positive cells following a standard gating protocol (Supplemental Figure 5). Alveolar epithelial cells were identified as EpCam-PerCP-Cy5.5–positive cells.

### Flow cytometry analysis of LIP uptake by heart, spleen, and liver cells.

Mice were I.N. instilled with 80 μg rhodamine-B–labeled dextran 70 kDa (Thermo Fisher Scientific) complexed in LIPs (480 μg total) suspended in a surfactant-containing suspension (5 mg/mL). A total of 60 μL of LIP-surfactant suspension was instilled. Then, 2 hours later, we isolated heart, spleen, and liver cells. To isolate cells, we buffer (PBS, Ca^2+^, and Mg^2+^ free) perfused systemic circulation through vascular cannulas to clear blood; we then minced and passed the tissues through 40 μm cell strainers (BD Biosciences) to obtain single-cell suspensions. For flow cytometry, we surface-stained the cells from each organ by incubating the suspension with relevant antibodies away from direct light for 1 hour at room temperature. We analyzed cells by flow cytometry (5L Cytek Aurora) according to manufacturer’s protocols using standard software (FCS Express 7 Flow). LIPs were identified as rhodamine B–positive events following a standard gating protocol (Supplemental Figure 4).

### Participants.

Confocal microscopy was done with intact human lungs obtained from brain-dead organ donors at the time of tissue acquisition for transplantation and when not used for clinical transplant, as described (9), through collaboration and protocol with LiveOnNY, the organ procurement organization for the New York area. Demographic data are detailed in Supplemental Table 2.

### Privacy protection.

None of the investigators had access to identifiable private information, and all samples were assigned unique, nonidentifying IDs on receipt. Their correspondence to the United Network for Organ Sharing IDs assigned by the organ procurement organizations (OPOs) and LiveOnNY was not communicated to the OPOs by the investigators, and the investigators maintained them under a secure, password-protected network.

### Statistics.

All groups comprised a minimum of 3 mice each. Group numbers were designed to enable detection of statistically significant differences with a power of at least 85%. For applicable imaging experiments, we carried out a paired protocol, where baseline and test conditions were obtained in the same alveolus or cell, and at least 3 determinations were obtained per lung. These determinations were averaged to obtain a mean for each condition in each lung. The means for each lung were pooled for the group to obtain mean ± SEM, where *n* represents the number of lungs unless otherwise stated. Data are shown as mean ± SEM. We analyzed data using the 2-tailed Student’s *t* test. Significance was accepted at *P* < 0.05.

### Study approval.

Animal procedures were approved by the Institutional Animal Care and Use Committee of the Vagelos College of Physicians and Surgeons at Columbia University. As confirmed by the Columbia University IRB, per the NIH policy, since all human samples in the study were acquired from deceased individuals, the study was not considered human subject research. This policy is based on United States Department of Health and Human Services human subject regulations under 45 CFR 46.

### Data availability.

All data are available in the Supporting Data Values file.

## Author contributions

LM and JB conceived the study. LM, MNI, GAG, and BK developed methodology. LM and MNI performed investigation. LM and JB performed visualization. LM and JB acquired funding. LM and JB performed project administration. JB supervised. LM wrote the original draft. LM, MNI, GAG, BK, SB, and JB reviewed and edited the manuscript. The order of co–first authors was determined by the extent of author contribution.

## Funding support

This work is the result of NIH funding, in whole or in part, and is subject to the NIH Public Access Policy. Through acceptance of this federal funding, the NIH has been given a right to make the work publicly available in PubMed Central.

- National Institutes of Health grant HL36024 (JB).
- Department of Defense grant PR211516 (JB).
- American Heart Association Career Development Award 24CDA1263614 (LM).
- American Heart Association Postdoctoral Fellowship 902655 (LM).
- American Thoracic Society Unrestricted Research grant 22-23U3 (LM).
- National Institutes of Health grant HL175058 (MNI).
- National Institutes of Health award S10OD020056 (Columbia Center for Translational Immunology flow cytometry core).

## Supplementary Material

Supplemental data

Supporting data values

## Version 1 10/30/2025

In-Press Preview

## Version 2 12/08/2025

Electronic publication
