# Supplementary material for: Macrophage-specific therapy blocks the lung’s mechanosensitive immune response to alveolar distension
Source: JCI Insight. 2025 Oct 30;10(23):e191853. doi: 10.1172/jci.insight.191853 (PMC12890529; doi:10.1172/jci.insight.191853)
Supplement: Supplemental data [file jciinsight-10-191853-s094.pdf]

SUPPLEMENTAL FIGURE 1

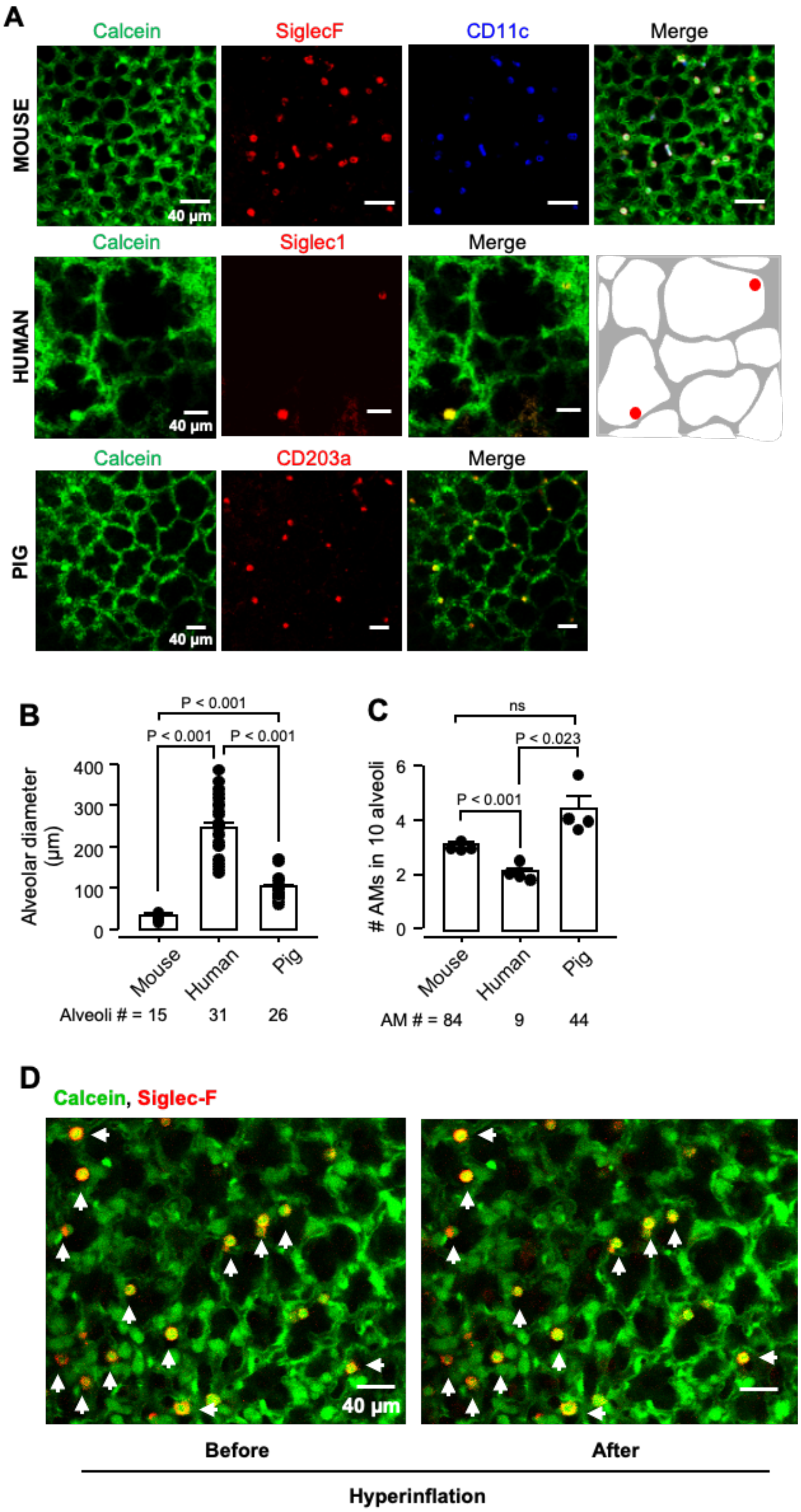

Supplemental Figure 1. Sessile AM real time confocal microscopy

A. Confocal images (*upper, middle and lower panels*) respectively, show calcein loaded alveoli from mouse, human and pig lung. AMs are identified by indicated markers. Sketch shows AM locations in an alveolar field.

B, C. Group data are for alveolar diameter (*B*) and the number of AMs in 10 alveoli (*C*) in 4 lungs each from mouse, human, and pig.

D. Confocal images show calcein loaded alveoli from a mouse lung. AMs are identified by SiglecF. Findings replicated at least 3 times in 3 lungs. AMs remained at the same locations before and after hyperinflation (arrowheads).

Data Analyses. Group data are mean±sem. Each dot represents data from one lung. P values were calculated by ANOVA with Bonferroni correction (B, C).

SUPPLEMENTAL FIGURE 2

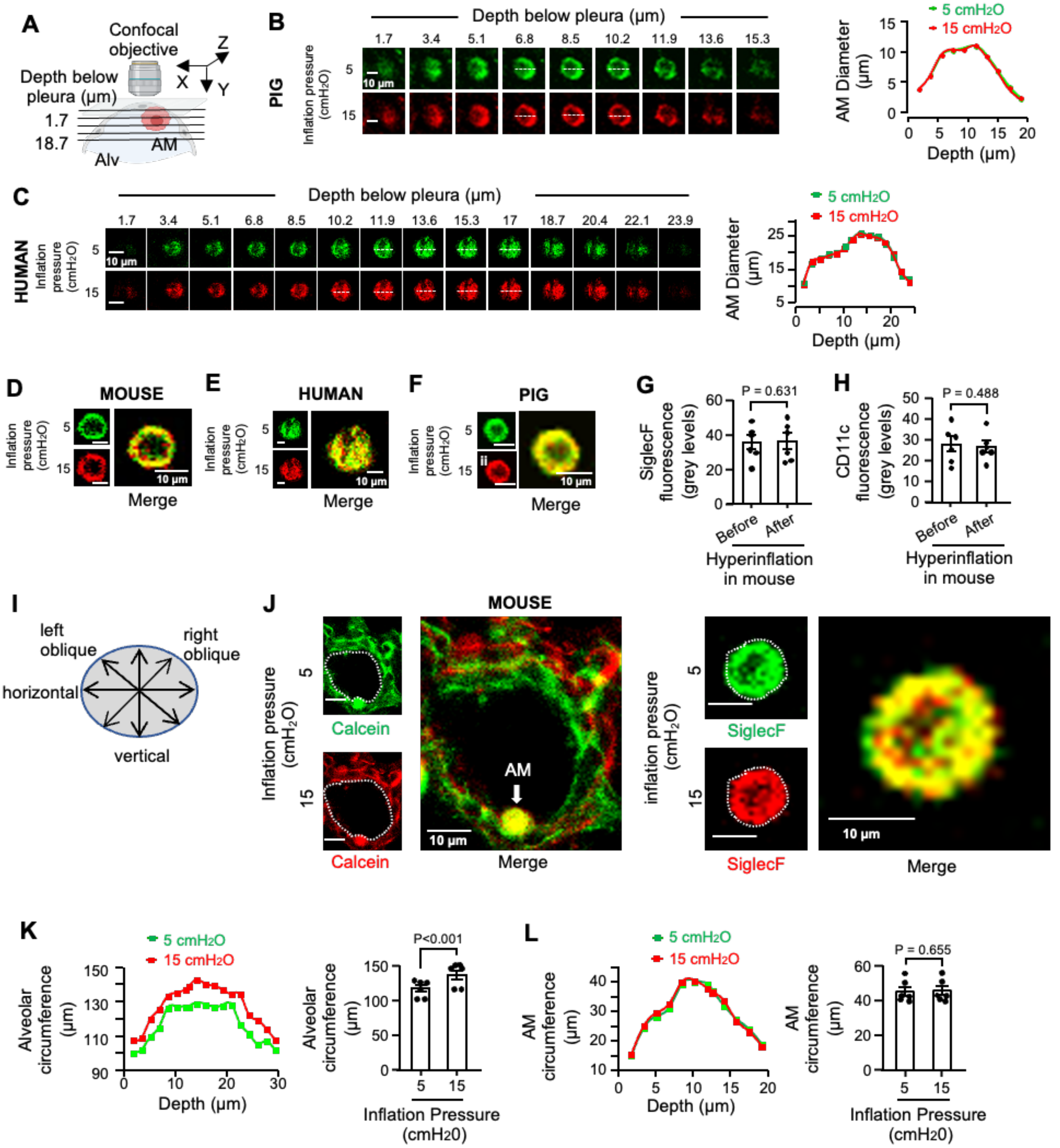

Supplemental Figure 2. Sessile AMs are strain-protected during alveolar stretch

A. Sketch shows the imaging protocol for *B*, *C*. Sketch was made using BioRender.

B, C. Confocal images show a single AM from pig (*B*) and human (*C*) lungs at high magnification. The AM immunofluorescence for CD203a (pig) and Siglec1 (human) are shown

in green and red pseudocolors for the indicated inflation pressures. The tracings plot AM diameter in the horizontal axis at different depths below the pleura. Findings replicated at least 3 times in 3 lungs for each species.

D-F. Confocal z stack images show mouse (*D*, SiglecF), human (*E*, Siglec1) and pig (*F*, CD203a) AM perimeter at 5 (green) and 15 (red) cmH_2O inflation pressure at different depths. Findings replicated at least 3 times in 3 lungs for each species.

G, H. Bars show quantifications of SiglecF (*G*) and CD11c (*H*) immunofluorescence in AMs (dots) before and after hyperinflation. n = 3 lungs for each bar.

I. Sketch shows AM diameter measuring protocol.

J. Representative confocal images show a mouse alveolus containing an AM (*left*) and the AM (*right*), respectively. Images were obtained at the indicated inflation pressures. The images are pseudocolored differently for the different inflation pressures. The merge images were obtained by superimposing the green and red images. The AM is located at the corner of the non-distending alveolar segment (*arrow*). The dotted lines indicate alveolar and AM circumference, respectively.

K, L. Tracings and group data are paired determinations of alveolar (*K*) and AM (*L*) circumference quantified at indicated inflation pressures. Dots in group data are lung averages.

Data Analyses. Group data are mean±sem. P values were calculated by paired t-test (G, H, K and L).

SUPPLEMENTAL FIGURE 3

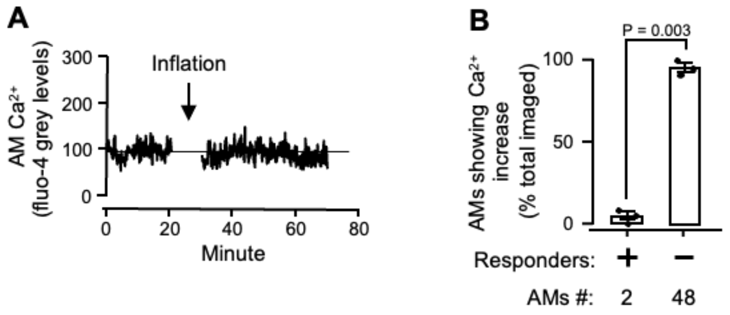

Supplemental Figure 3. Lack of Ca^{2+} increases in mouse AMs in response to inflation from 5 to 10 cmH_2O

A, B. Tracing (*A*) shows alveolar macrophage (*AM*) cytosolic calcium (Ca^{2+}) responses before and after an increase of inflation pressure from 5 to 10 cmH_2O. Black line indicates mean cytosolic calcium. Bars (*B*) show lack of AM responders to the increased inflation in 3 lungs for each bar. Group data are mean±sem, P value was calculated by unpaired t-test.

SUPPLEMENTAL FIGURE 4

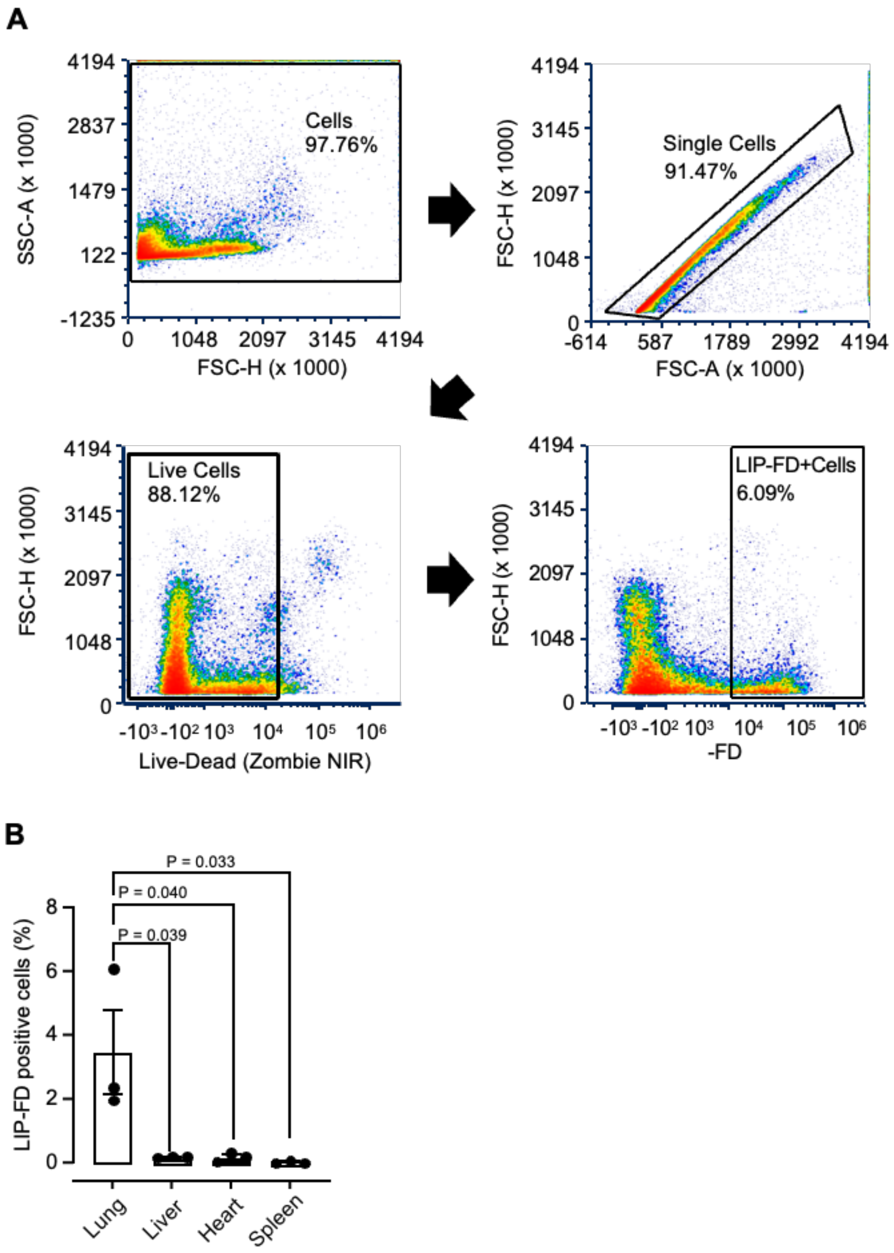

Supplemental Figure 4. Lack of LIP escape to peripheral organs

A. Gating strategy used to identify liposome-encapsulating fluorescent dextran 70 kDa (*LIP-FD)* positive cells from mouse lung instilled with LIP-FD 2 hours prior. Same gating strategy was used to identify LIP-FD cells from liver, heart and spleen tissue. Cells were isolated from organs and after the exclusion of dead cells, debris and doublets, LIP-FD cells were identified by LIP-FD staining.

B. Bars show percentage of LIP-FD positive cells in lung, liver, heart and spleen tissue from mice instilled with LIP-FD two hours prior. Group data are mean±sem, P value was calculated by unpaired t-test.

SUPPLEMENTAL FIGURE 5

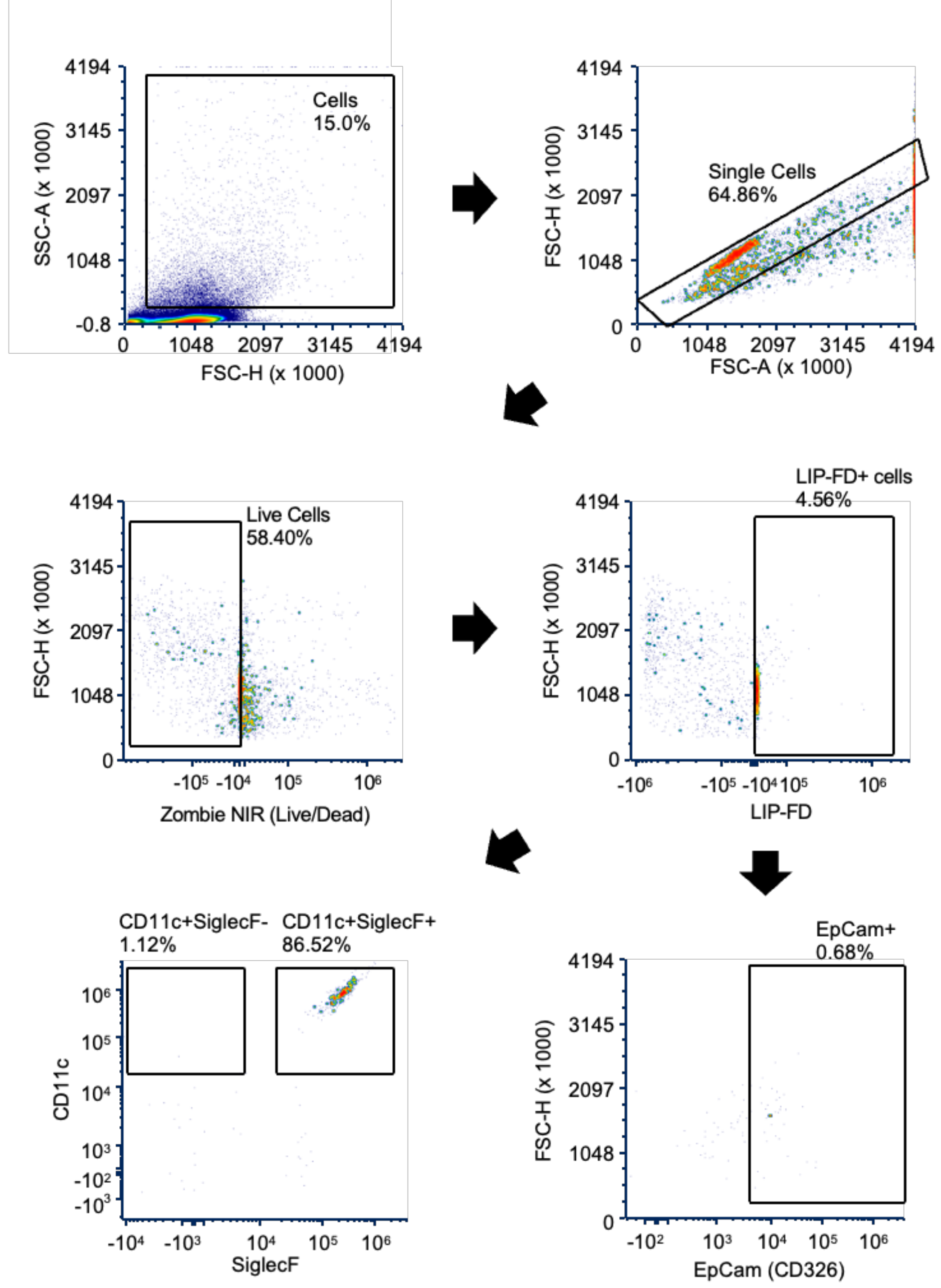

Supplemental Figure 5. Flow cytometry gating strategy to identify alveolar macrophages, dendritic cells and alveolar epithelium in lung homogenates

Gating strategy used to identify sessile AMs, DCs, and AE from mouse lung tissue homogenates**.** A mouse was intranasally instilled with liposome-encapsulated rhodamine b dextran 70 kDa LIPs (*LIP-FD*). Two hours later, cells were isolated from lung homogenates and after the exclusion of debris and doublets. Cells positive for LIP-FD were identified. CD11c-APC and SiglecF-488 and EpCam-PerCP-Cy5.5 were used to identify alveolar macrophages and alveolar epithelium, respectively.

SUPPLEMENTAL FIGURE 6

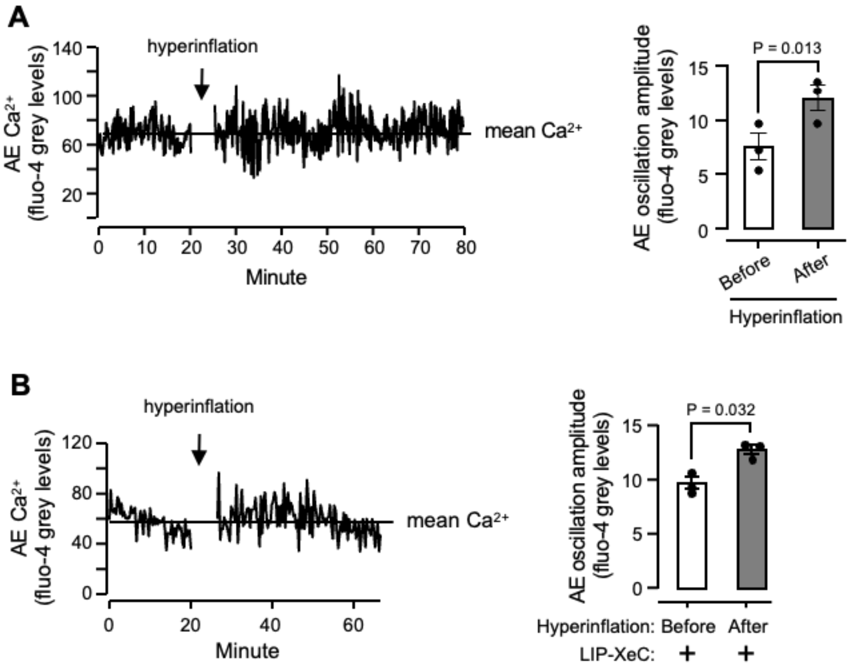

Supplemental Figure 6. Hyperinflation-induced increases in alveolar epithelial Ca^{2+} oscillations in lungs treated with or without LIP-XeC

A. Tracing shows alveolar epithelial (AE) cytosolic calcium (Ca^{2+}) responses before and after hyperinflation. Black line indicates mean cytosolic calcium. Bars show paired quantifications of AE Ca^{2+} oscillation amplitude in AE (dots) before and after hyperinflation.

B. Tracing shows alveolar epithelial (AE) cytosolic calcium (Ca^{2+}) responses before and after hyperinflation in lungs instilled with liposome-encapsulating Xestospongin C (LIP-XeC) 2 hours prior to experiment. Black line indicates mean cytosolic calcium. Bars show paired quantifications of AE Ca^{2+} oscillation amplitude in AE (dots) before and after hyperinflation.

Data Analyses. Group data are mean±sem. P values were calculated by paired t-test (A, B).

SUPPLEMENTAL FIGURE 7

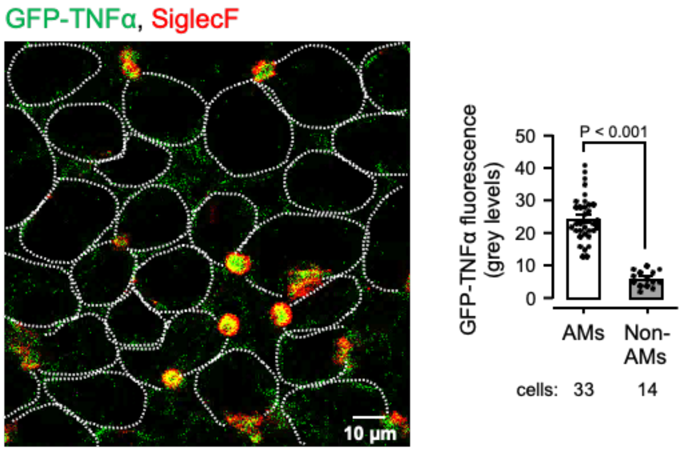

Supplemental Figure 7. GFP-TNFα fluorescence in sessile AMs

Confocal image shows a field of AMs which were transfected with liposomes containing GFP-TNFα plasmid in a surfactant containing solution by intranasal instillation two hours prior. White dashed lines indicate alveolar perimeters. Bars show that majority of GFP-TNFα fluorescence was expressed in imaged sessile AMs compared to randomly selected non-AM (SiglecF negative) regions. Group data are mean±sem, findings replicated in 3 lungs, P value was calculated by unpaired t-test.

SUPPLEMENTAL FIGURE 8

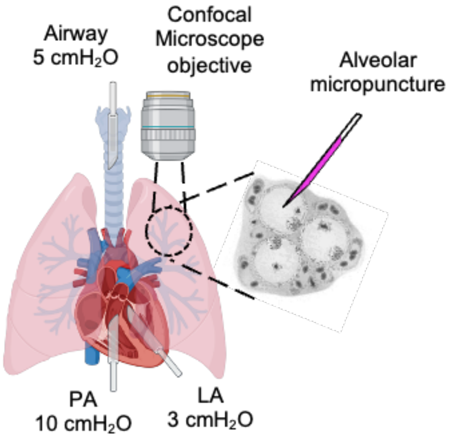

Supplemental Figure 8. Isolated, perfused lung preparation and alveolar micropuncture technique.

Schematic of isolated, perfused lung preparation and alveolar micropuncture technique to deliver fluorescent agents that permit visualization of the alveolar microenvironment under confocal microscopy. *PA*, pulmonary artery. *LA*, left atrium. Sketch was made using Biorender.

SUPPLEMENTAL FIGURE 9

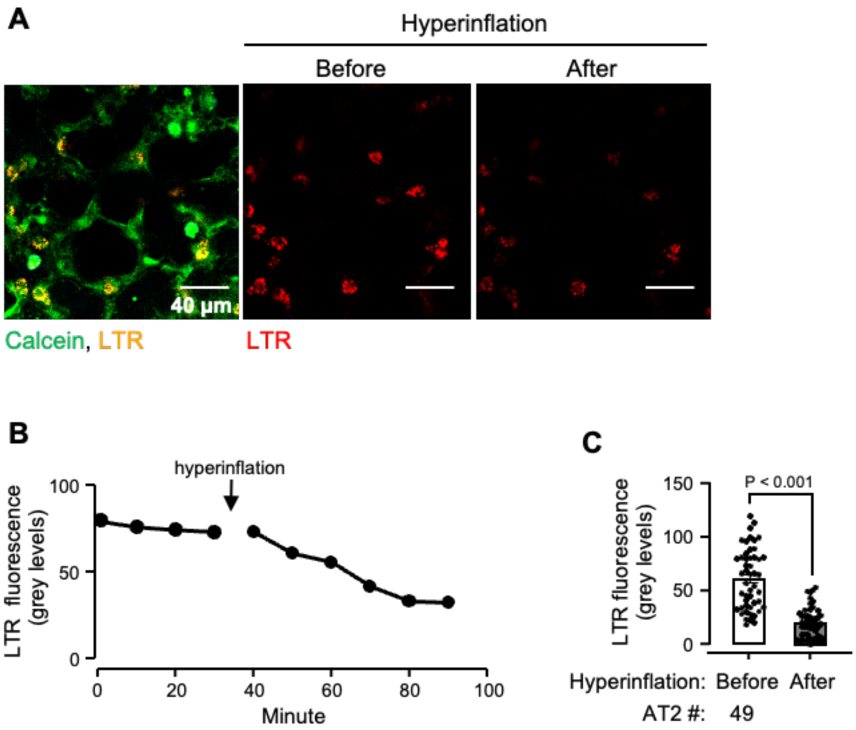

Supplemental Figure 9. Hyperinflation-induced secretion of surfactant

A. Confocal image shows epithelial fluorescence in alveolar epithelium infused with calcein (*green*) and lysotracker red (LTR) (*red*). Confocal images show LTR fluorescence before (*middle*) and 60 min after (*right*) hyperinflation.

B. Tracings from a single LTR+ cell show LTR fluorescence before and after hyperinflation at indicated times.

C. Bars show paired quantifications of LTR fluorescence (dots) before and 60 min after hyperinflation. Group data are mean±sem, n = 49 alveolar epithelial type 2 cells from 4 lungs, P value was calculated by paired t-test.

SUPPLEMENTAL TABLE 1

Name, Fluorescent Dye	Supplier	Catalogue No.	Stock Conc.	Final Conc.	Lot No.
CD45, Pacific Blue	Biolegend	103126	0.5 mg/ml	5 µg/ml	B314223
Ly-6G, PE-Cy7	Biolegend	127618	0.2 mg/ml	2 µg/ml	B351626
CD11b, PE-Cy5	Biolegend	101210	0.2 mg/ml	2 µg/ml	B260953
CD11c, APC	Biolegend	117310	0.2 mg/ml	2 µg/ml	B278343
SiglecF, PE	Biolegend	155506	0.2 mg/ml	2 µg/ml	B301117I
Siglec1 (CD169)	Thermo Fisher	PA5-84155	0.17 mg/ml	3.4 µg/ml	ZA4189480
CD203a, Alexa 647	BioRad	MCA1973A647	0.05 mg/ml	5 µg/ml	152566
Goat anti-rabbit IgG, Alexa Fluor 488)	Molecular Probes	A-11008	1 mg/ml	100 µg/ml	2743033
SiglecF, Alexa Fluor 488	Invitrogen	53-1702-80	0.2 mg/ml	2 µg/ml	2072850
EpCam, PerCP-Cy5.5	Biologend	118219	0.2 mg/ml	2 µg/ml	B406473
TNFR1, Alexa Fluor 633	AbD Serotec	MCA2350	10 mg/ml	40 µg/ml	n/a

Supplemental Table 1. Table of antibodies used in imaging and flow cytometry studies

Final concentration (conc.) indicates concentration used in experiments.

SUPPLEMENTAL TABLE 2

Donor Network	Donor	Age	Gender	Smoking	Substance use	Ethnicity
LiveOnNY	D536	34	F	Y	Marijuana, Amphetamines, PCP, Cocaine, and Barbituates	Hispanic/Latino
LiveOnNY	D546	52	M	Y	THC, Cocaine, Opiates	Hispanic/Latino
LiveOnNY	D567	46	M	Y	Cocaine, EtOH	Hispanic/Latino
LiveOnNY	D672	82	M	Y	EtOH	Hispanic/Latino

Supplemental Table 2. Participants

Demographic data for each donor is indicated.
